# Pathogenic Potential of *Saccharomyces* Strains Isolated from Dietary Supplements

**DOI:** 10.1371/journal.pone.0098094

**Published:** 2014-05-30

**Authors:** Silvia Llopis, Carolina Hernández-Haro, Lucía Monteoliva, Amparo Querol, María Molina, María T. Fernández-Espinar

**Affiliations:** 1 Departamento de Biotecnología de los Alimentos, Instituto de Agroquímica y Tecnología de Alimentos (CSIC), Paterna, Valencia, Spain; 2 Departamento de Microbiología II, Facultad de Farmacia, Universidad Complutense de Madrid and Instituto Ramón y Cajal de Investigación Sanitaria (IRYCIS), Madrid, Spain; Louisiana State University, United States of America

## Abstract

*Saccharomyces cerevisiae* plays a beneficial role in health because of its intrinsic nutritional value and bio-functional properties, which is why it is also used as a dietary supplement. However, the perception that *S. cerevisiae* is harmless has changed due to an increasing number of infections caused by this yeast. Given this scenario, we have tested whether viable strains contained in dietary supplements displayed virulence-associated phenotypic traits that could contribute to virulence in humans. We have also performed an *in vivo* study of the pathogenic potential of these strains using a murine model of systemic infection by intravenous inoculation. A total of 5 strains were isolated from 22 commercial products and tested. Results highlight one strain (D14) in terms of burden levels in brains and kidneys and ability to cause death, whereas the other two strains (D2 and D4) were considered of low virulence. Our results suggest a strong relationship between some of the virulence-associated phenotypic traits (ability to grow at 39°C and pseudohyphal growth) and the *in vivo* virulence in a mouse model of intravenous inoculation for isolates under study. The isolate displaying greatest virulence (D14) was evaluated in an experimental murine model of gastrointestinal infection with immunosuppression and disruption of mucosal integrity, which are common risk factors for developing infection in humans, and results were compared with an avirulent strain (D23). We showed that D14 was able to spread to mesenteric nodes and distant organs under these conditions. Given the widespread consumption of dietary supplements, we recommend only safe strains be used.

## Introduction

The yeast *Saccharomyces cerevisiae* is well known mainly for its use in the production of foods (bread) and of alcoholic beverages (wines, beers, ciders, saké) through alcoholic fermentation. In addition to its use in fermentative processes, *S. cerevisiae* is outstanding for its intrinsic nutritional value, a reason why it is also used in dietary supplements. Moreover, these preparations are believed to possess some bio-functional properties that exert positive effects on one or more of the host’s physiological functions due to its content in minerals and B-complex vitamins. The possible beneficial effects of consumption are well known [1] and there are studies into its medicinal properties [2], [3], [4], [5]. This is why the consumption of nutritional yeasts is a current trend promoted by healthy diets and lifestyles.

Although the bio-functional properties do not depend on whether yeast cells are alive (the majority of commercial products contain deactivated cells), some companies include revivifying cells in their products, as indicated on the labels. In these cases, living cells enter the body in ongoing and high concentrations (an average daily intake of 2.1×10^7^ cells is recommended). Despite this growing trend, there has not been a great deal of interest in the possible undesirable effects of consumption. This is mainly because *S. cerevisiae* has always been considered a safe micro-organism for nutritional use (“GRAS”, Generally Regarded as Safe) without considering its undesirable facets. However, this concept is currently changing due to the increasing incidence of infections associated with this yeast species; a comprehensive review of the topic shows that from 92 documented cases of invasive *Saccharomyces* infection, 15 were diagnosed before 1990, while 76 cases were diagnosed after 1990 [6].

*Saccharomyces cerevisiae* fungemia in humans mainly occurs in immunodepressed patients. Nevertheless, the existence of strains with inherent virulent potential cannot be ruled out since, although it is not common, cases of fungemia by this yeast have also been described in healthy hosts [7], [8], [9]. The few studies assessing the pathogenic potential of this yeast species are indicative of the aforementioned risk, and show that most clinical isolates of *S. cerevisiae* exhibit certain phenotypic characteristics [10], [11], [12], [13], [14] and display different degrees of virulence when tested in murine models [15], [16], [17]. These phenotypic traits include growth at 42°C, some hydrolytic activities, pseudohyphal and invasive growth, switching and adhesion, which have been associated with virulence in pathogenic microorganisms [18]–[27]. Some of these traits have been studied more recently in our laboratory in a large number of clinical and non-clinical isolates [28]. Activation of signal transduction pathways mediated by the Mitogen-Activated Protein Kinases (MAPK) Kss1, which regulates pseudohyphal and invasive growth, and Slt2 from the cell wall integrity pathway (CWI) [29], have been also analyzed in these strains [30]. Interestingly, we observed that a commercial baker’s yeast as well as *S. cerevisiae* var. *boulardii* (strain isolated from a commercial lot of the bio-therapeutic agent Ultra-Levura) appeared to be related to clinical strains based on such phenotypic traits [28], and they presented remarkable dissemination capacity in murine models of systemic infection [31]. Furthermore, they were encountered in the blood of individuals with symptoms of infection (baker’s yeast [32], *S. cerevisiae* var. *boulardii* [33], [34], [35]). Given these considerations one might ask whether the ingestion of live *S. cerevisiae* cells, through the consumption of these products, can cause infection in humans. Indeed, a case of fever of unknown origin was subsequently associated with the prolonged ingestion of a dietary supplement containing between 10^7^ and 10^8^ of *S. cerevisiae* per gram of product [36], which suggests that such doubts may be founded. In addition, previous results obtained in our laboratory showed that virulence in two murine models of a strain (named D14) from a commercial dietary supplement was notable compared with a group of clinical isolates [37].

Since dietary supplements are a route of entry by which live *S. cerevisiae* cells enter the human body, and given there are no safety studies to date, we undertook an in-depth study of the virulence of the aforementioned isolate D14, and other isolates from commercial products. We first identified the isolates molecularly to verify their adscription to the *S. cerevisiae* species since misidentified strains are found quite frequently [38]. Moreover, we studied whether hybrid strains between *S. cerevisiae* and other closely related *Saccharomyces* species are present in yeast-enriched foods and dietary products. In fermentative wine processes, several wine *Saccharomyces* strains considered to be *S. cerevisiae* have been reported to be hybrids [39], [40]. Hybrid generation could provide adaptive values [41] hence it is interesting to evaluate whether this affects the development of infection in mammalian hosts. We analyzed whether the isolates exhibited phenotypic traits (in particular, growth at high temperatures, secretion of hydrolytic enzymes, pseudohyphal and invasive growth, colony phenotype switching, MAPK activation and plastic adherence) associated with virulence in other pathogenic yeasts before evaluating their virulence *in vivo*. We then studied the potentially virulent strains (those with the highest percentage of positive traits) in a murine model. To do so, we evaluated death rates and determined burdens in brains and kidneys in immunocompetent BALB/c after intravenous inoculation and compared them with an isolate displaying fewer positive traits. Finally, and taking into account that the natural portal of entry of live yeast cells is oral intake, we tested a murine model of disseminated infection derived from translocation across the gastrointestinal mucosa to determine whether the strains with invasive infection ability pose a real potential safety risk. Given the complexity of such murine oral inoculation models, we tested only the isolates showing the highest and the lowest virulence.

## Materials and Methods

### Strains

Yeast strains used for the elaboration of commercial dietary products were isolated from 22 products (D1–D4 and D6–D23), listed in Table 1. These products included two enriched beers and 20 dietary products. *S. cerevisiae* strains used as control in the *in vitro* study of virulence-associated phenotypes and in the *in vivo* virulence study by intravenous inoculation (i.v.) in mice are listed in Table 2. The *Candida albicans* SC5314 strain [42] was used as positive control in the assay of adherence to plastics.

**Table 1:** Commercial products analyzed in this study.

Product code	Commercial name	Presentation	Company
D1	“Levadura de cerveza y germen de trigo”(a)	Pills	ALTANA PHARMA, S.A.
D2	Levivel	Pills	NOVA DIET, S.A.
D3	“Levadura de cerveza”(b) (Arkocaps)	Pills	ARKOPHARMA
D4	“Levadura”(c) A+E	Pills	ALTANA PHARMA, S.A.
D5	“Ultra-Levura” (*S. cerevisiae var. boulardii*)	Pills	UPSAMEDICA, S.L.
D6	“Levadura de cerveza”(b)	Liquid	INSTITUTO FERRÁN
D7	Levadiet	Pills	DIETISA
D8	“Levadura de cerveza”(b)	Flakes	TERRA VERDA
D9	“Levadura de cerveza”(b)	Pills	TERRA VERDA
D10	Bioland beer	Liquid	BRAUEREI SCHLESWIG
D11	Panaktiv	Liquid	Dr. H. Metz
D12	“Levadura y germen”(d)	Pills	SOTYA, S.A.
D13	Strath	Pills	DIETISA
D14	Phytodepur	Pills	Dietéticos INTERSA, S.A.
D15	“Levadura copos”(e) Bioreal	Flakes	RAPUNZEL
D16	“Levadura viva + selenio”(f)	Pills	NATURLIFE, S.L.
D17	Depurator	Liquid	INTERSA, S.A.
D18	Levatrig	Pills	BIOSERUM Laboratorios S.L.
D19	“Levadura de cerveza”(b)	Pills	Distribuciones FELIU
D20	Phytonorm	Pills	INTERSA, S.A.
D21	“Levadura de cerveza”(b)	Flakes	Int-Salim (SALUD E IMAGINACIÓN, S.L.)
D22	Levadiet-C	Flakes	DIETISA
D23	“Cerveza biológica”(g) PILS	Liquid	RAPUNZEL

(a) Brewer’s yeast and wheat germ;

(b) Brewer’s yeast;

(c) Yeast;

(d) Yeast and germ;

(e) Yeast flakes;

(f) Yeast alive;

(g) Biological beer.

**Table 2:** *S. cerevisiae* strains used as control and assays for which they were used.

Strain	Origin	Reportedvirulence-associatedphenotypes	Reported*in vivo*virulence	Reference	Use in this work
“Ultra-Levura”**^(a,b)^**	Commercial	Growth at 42°C, proteaseand phospholipasesecretion andPh growth	Moderatelyvirulent	[17], [28], [31]	Control in the *in vitro* **^(c,d,e^** ^,**f)**^ study
102(b)	Respiratory	Growth at 42°C, proteaseand phospholipasesecretion, Ph and invasivegrowth	Virulent	[28], [31]	Control in the *in vitro* **^(c,f)^** study
CECT 10.431	Wine	Low invasivegrowth	Non-virulent	[28], [31]	Control in the *in vitro* **^(c,d)^** and *in vivo* (g) studies
YJM128	Lung	Growth at42°C, proteasesecretion, Phand invasivegrowth	Virulent	[16]	Control in the *in vivo* (g) study
W303(h)	Laboratory	n.r.	n.r.	-	Control in the *in vitro* **^(c,d,f)^** and *in vivo* (g) studies
BY4741(i)	Laboratory	n.r.	n.r.	-	Control in the *in vitro* **^(e,f)^** study

(a) (named D5 in Table 1);

(b) Molecular characterization of these strains have been described previously [44];

(c) Growth at different temperatures,

(d) protease and phospholipase secretion, Ph (pseudohyphal) and invasive growth;

(e) MAPK activation;

(f) Adherence to plastics;

(g) *In vivo* study by intravenous inoculation of strains in BALB/c mice;

(h) (*MAT* **a** *; ura3-52; trp1D2; leu2-3_112; his3-11; ade2-1; can1-100*) [64], [76];

(i) (*MAT* **a** *his3*Δ*1 leu2*Δ*0 met15*Δ*0 ura3*Δ*0)* (EUROSCARF); n.r.: Non reported.

### Growth Conditions

For dietetic strains isolation, a sample from each commercial product (1 pill, 1 ml or a teaspoonful depending on whether the product was in pill, liquid of flake form) was grown in 4.5 ml of YPD liquid medium [1% (w/v) yeast extract (Pronadisa, Madrid, Spain), 2% (w/v) peptone (Oxoid LTD, Basingstoke, England), 2% (w/v) glucose (Panreac, Barcelona, Spain)] two days at 30°C. Then, 50 µl of each culture were spread onto YPD plates [YPD medium+2% w/v agar (Panreac, Barcelona, Spain)] and incubated 24–48 h at 30°C. A total of six colonies, from each of the products that exhibited yeast growth, were randomly chosen for further identification and characterization.

For infection experiments *Saccharomyces* strains were grown at 30°C on YPD plates. After 24 h, cells were harvested, washed twice with sterile phosphate-buffered saline (PBS) and diluted to the desired concentration in the same buffer for intravenous injection or oral administration.

### PCR Amplification and Enzymatic Restriction

DNA was isolated according to Querol *et al*. [43] with the modifications described by de Llanos *et al*. [44].

For PCR amplification, DNA was diluted to 1–50 ng/µl. The amplification of the ribosomal 5.8S-ITS region was carried out with primers its1 and its4 [45] under the reaction and amplification conditions described previously by de Llanos *et al*. [44]. PCR products (15 µl) were digested without further purification with the restriction endonucleases *Acc*I, *Cfo*I, *Hae*III and *Hin*fI (Boehringer Mannhein, Germany). Restriction pattern comparisons were performed using the Yeast-id database (http://www.yeast-id.org).

*Saccharomyces* interspecific hybrids were identified by PCR amplification of three nuclear genes *APM3, PEX2* and *BRE5* located in different chromosomes (II, X and XIV, respectively) and their subsequent RFLP using the endonucleases *Msp*I, *Hae*III and *Hin*fI (Boehringer Mannhein, Germany) respectively. PCR amplification conditions and pair of primers were those described previously by González *et al*. [39]. Characterization was performed by comparing the restriction patterns obtained with those described in previous works for type reference strains of the species *Saccharomyces uvarum*, *S. cerevisiae* and *S. kudriavzevii* [39], [46].

δ sequences were amplified using primers δ1 and δ2 as previously described by Ness *et al.* [47] and amplification conditions were according to de Llanos *et al*. [44]. PCR products and restriction fragments were visualized on 1.4% and 3% agarose (Pronadisa; Laboratorios Conda S.A., Madrid, Spain) gels, respectively. A 100-bp DNA ladder marker (Invitrogen Life Technologies) served as a size standard.

For mtDNA restriction analysis, DNA was resuspended in 13 µl of water and used in full in the reaction. The restriction analysis was performed according to Querol *et al*. [43]. DNA was digested with *Hin*fI (Boehringer Mannhein, Germany), following the supplier’s instructions. Restriction fragments were visualized on 0.8% agarose (Pronadisa; Laboratorios Conda S.A., Madrid, Spain) gels. The DNA of phage λ digested with *Pst*I served as size standard.

### Virulence-associated Phenotypes of Yeasts Isolated from Commercial Products

#### Growth at different temperatures

Yeast strains were cultured in YPD liquid medium at 30°C overnight. Yeast suspensions were adjusted to 0.5 OD at 600 nm, corresponding to 5×10^6^ cells/ml; serial decimal dilutions were performed and 5 µl of them were spotted (in triplicate) on YPD plates and incubated at 30, 37, 39 or 42°C for 1–3 days.

To determine generation time, strains were cultured in liquid YPD at 30°C and 37°C. Absorbance of the cultures was measured at 600 nm every hour for 8 h. Linear regression was used to calculate specific growth rate and generation time. The statistic t-Test was performed on these data.

#### Secretion of hydrolytic enzymes (protease and phospholipase)

Protease production was determined according to Aoki *et al*. [48]. The test medium consisted of agar plates containing bovine serum albumin (BSA): 60 ml of solution was prepared containing 0.04 g MgSO_4_. 7H_2_O (Panreac, Barcelona, Spain), 0.5 g K_2_HPO_4_ (Panreac, Barcelona, Spain), 1 g NaCl (Panreac, Barcelona, Spain), 0.2 g dried yeast extract (Pronadisa, Madrid, Spain), 4 g glucose (Panreac, Barcelona, Spain) and 0.5 g BSA (Fraction V, Sigma, USA), the pH was adjusted to 5 with 1N HCl. The solution was sterilized by filtration and mixed with 140 ml of melted agar, 20 ml of this medium was poured into each Petri dish and 5 µl of a 30°C overnight yeast culture adjusted to 0.3–0.4 OD at 600 nm were inoculated in each Petri dish and incubated at 37°C for 4 days.

Phospholipase production was determined with Egg-Yolk medium used to detect lipolytic activity [49]. This medium consisted of Sabouraud Dextrose Agar (Difco), NaCl 11.7 g (Panreac, Barcelona, Spain); CaCl_2_ 0.111 g (Panreac, Barcelona, Spain); and 10% (v/v) sterile egg yolk (Pronadisa, Madrid, Spain) in 184 ml distilled water. The plates were inoculated with 5 µl of a 30°C overnight yeast culture adjusted to 0.3–0.4 OD at 600 nm and incubated at 30°C for 7–10 days.

In both assays, activity was visualized as an area of precipitation around each colony. The value of enzymatic activity (Pz) was measured according to the method of Price *et al.* [49] and was expressed as the ratio of the diameter of the colony alone to the diameter of the colony plus the precipitation zone; thus a low Pz indicated high enzyme production. The average Pz value was obtained using three separate samples of each strain.

#### Pseudohyphal and Invasive growth

Synthetic low ammonia dextrose media [SLAD: 0.67% of yeast nitrogen base (YNB) without amino acids and ammonium sulfate (Difco), 0.05 mM ammonium sulfate (Sigma-Aldrich) as sole nitrogen source, 2% anhydrous D-glucose (Panreac, Barcelona, Spain), 2% of washed agar] was used to assay pseudohyphal growth [50]. Strains were streaked on SLAD plates and then observed after four days of growth at 30°C.

To detect invasiveness, strains were patched carefully onto YPD plates to avoid scratching the agar, incubated at 30°C for three days, and then at room temperature for two additional days. A gentle stream of deionized water was then used to rinse all of the cells from the agar surface in order to observe the presence of cells growing below the agar surface.

#### Colony phenotype switching

Yeast strains were cultured in YPD liquid medium at 30°C overnight. Cells were collected by centrifugation and resuspended in sterile water to a concentration of 10^3^ cells/ml, and 100 µl of this suspension was spread onto YPD agar plates with 1% phloxine B (Sigma-Aldrich) [12]. Plates were incubated for five days at 30°C and colonial phenotypes were then observed and counted. Stability and reversion were studied for the most abundant secondary phenotypes by the assay described above.

#### Western blotting assays

Yeast cells were cultured in YPD liquid medium at 24°C or 39°C. Cell collection, protein extracts, protein separation by SDS–PAGE and electroblotting to nitrocellulose membranes were carried out as previously described [51]. Polyclonal anti-phospho-p44/p42 MAPK (Thr-202/Tyr-204) antibody (Cell Signalling Technology) was used to detect dually phosphorylated Slt2 and Kss1. Total amounts of Slt2, Kss1 and actin were detected using polyclonal anti-GST-Slt2 [52], polyclonal anti-Kss1 (Santa Cruz Biotechnology) and monoclonal C4 anti-actin (ICN Biomedicals) antibodies, respectively. Species-appropriate fluorescently conjugated secondary antibodies were used. Membranes were analyzed with Odyssey Infrared Imaging System (LI-COR Biosciences).

#### Adherence to polystyrene

To determine adherence to polystyrene Petri plates, yeast strains were cultured in liquid YPD at 30°C overnight. Cells were collected by centrifugation and resuspended in glucosaline solution (0.2 M D-glucose, 0.05 M NaCl) to reach a 10^6^ cells/ml concentration. Ten ml of each cell suspension were added to polystyrene Petri plates of 90 mm of diameter (J. D. Catalan, S.L., Spain) and to glass tubes as control of no plastic adhesion, and incubated for 1 h at 37°C with 5% CO_2_. Supernatants containing non-adhered yeast cells were collected. Plates were washed twice with 5 ml of sterile water and washings were added to the collected suspension. These yeast cell suspensions were plated on YPD and incubated for two days at 30°C. Colonies were then counted and the difference between plate and glass tube counts was considered as the number of adherent cells.

To measure adherence to polystyrene 96-well microtiter plates, we used the protocol described by Peeters *et al.* [53] with some modifications. Yeast strains were cultured in liquid YPD at 30°C overnight. Cells were collected by centrifugation and resuspended in glucosaline solution (0.2 M Glucose, 0.05 M NaCl) to reach a 10^8^ cells/ml concentration. One–hundred µl of each suspension were inoculated into 16 wells of a 96-well polystyrene cell culture microplate (Greiner Bio-one) and 16 wells were filled with sterile glucosaline solution as negative controls. Microtiter plates were incubated for 1 h at 37°C with 5% CO_2_. Supernatant containing non-adhered cells was removed, wells were rinsed with glucosaline solution, and cells adhering to the wells were fixed with 99% methanol (15 min) and stained with 0.02% crystal violet solution (Panreac, Barcelona, Spain) for 20 min. Plates were washed to remove the excess of crystal violet. Finally, crystal violet from stained adhered yeast cells was extracted with 33% acetic acid, and absorbance measured at 595 nm.

#### Adherence to polyurethane intravenous catheters

Multi-lumen central venous catheterization sets (Arrow International) were used. Catheters were cut into 1 cm-long sections under sterile conditions. Yeast strains were cultured in liquid YPD at 30°C overnight. Cells were collected by centrifugation and suspensions containing 10^7^ cells/ml on either glucosaline solution or RPMI-1640 medium (Lonza) were obtained. One ml of each suspension was added to an eppendorf tube containing a catheter section. Eppendorf tubes were incubated for 1 and 24 h at 37°C in a rotating device. Catheter sections were then placed in new tubes and washed five times with sterile water. In each washing, tubes were gently inverted 10 times and water was removed by a glass Pasteur pipette connected to a vacuum pump. Then, each catheter section was transferred to a tube and 1 ml of sterile water was added. Each tube was shaken using a Vortex mixer for 10 minutes. Yeast suspensions were spread onto YPD plates and incubated two days at 30°C and then, colonies were counted.

### Mouse Model of Systemic Infection by Intravenous Inoculation

#### Animals

Two to four batches of 10 mice belonging to co-sanguineous strain BALB/c (immunocompetent) were used per *Saccharomyces* strain. All animals were obtained from Harlan Ibérica (Barcelona, Spain) and were 6-week-old female mice. On arrival, animals were left for between 4 to 5 days to acclimatize and provided water and food *ad libitum*.

Animal studies were carried out at the animal unit of the Central Support Service to Experimental Research of the University of Valencia (School of Pharmacy, University of Valencia, Spain). Animal manipulation was carried out in compliance with Spanish laws and guidelines (RD1201/2005 and law 32/2007) and according to the European Directive 2010/63/EU concerning the protection of animals used for scientific purposes. All the assays were approved by the Consejo Superior de Investigaciones Científicas (CSIC, Spain) Bioethics Committee.

#### Experimental infections

Mice were infected by intravenous (i.v.) inoculation into the tail vein with 2×10^7^ viable CFU of yeast in a volume of 0.2 ml of PBS, as previously described [16] and were sacrificed by cervical dislocation to determine yeast burdens in brains and kidneys at different time points as follows. On day +7, between two and four mice per batch were sacrificed except when death was detected prior day 7, in which case only one mouse was sacrificed reserving the surviving mice to determine burdens at days +15 and +30. The number of mice sacrificed per strain at these time points varied from two to four per batch. A control mouse was intravenously inoculated with 0.2 ml of sterile PBS alone. Organs were processed as described below.

### Mouse Model of Systemic Infection by Oral Inoculation

#### Animals

Twelve mice belonging to strain ICR/Swiss (outbread immuno-competent) were used per *Saccharomyces* strain. All animals were obtained from Harlan Ibérica (Barcelona, Spain) and were 5–6-week-old female mice. On arrival, animals were left for 10 days to acclimatize and provided water and food *ad libitum*. Animals were caged using sterilized bedding and provided sterilized food and water from the first day of antibiotic administration.

Animal studies were carried out at the University of Valencia mentioned above.

#### Experimental infection

To deplete the indigenous gastrointestinal microbiota and facilitate the presence of yeast in the gastrointestinal tract, mice were fed for five days prior to oral inoculation with sterile water containing three antibiotics as described for *Candida glabrata* [54]: 1 mg/ml bacitracin (Sigma-Aldrich Química, S.A. Madrid, Spain), 2 mg/ml streptomycin sulfate (Reig Jofré Laboratories, S.A. Barcelona) and 0.1 mg/ml gentamicin sulfate (Normon Laboratories, S.A. Madrid). The antibiotics were administered until the end of the experiment to reduce secondary infections. To assess antibiotic effectiveness, stools were collected during three consecutive days prior to oral inoculation (at days −3, −2 and −1) and at days 1, 3, 5 and 7.

A single dose of 0.2 ml of an inoculum containing 1×10^9^ of live yeast cells was administered to mice by intragastric intubation (day 0). In addition, the same strain was added to sterile water with the aforementioned antibiotics at approximately 7×10^9^ cfu/ml and was administered via the drinking water to mice for three consecutive days (days 0–2). Then, the inoculum was removed and replaced by drinking water with antibiotics for the remainder of the experiment.

#### Immunosuppression

Three days after the beginning of yeast administration, mice were given the immunosuppressant drug dexamethasone (Fortecortín 40 mg, Merck Farma y Química, S.L. Madrid, Spain) at 0.1 mg/g by intraperitoneally (ip) injection twice a day during four consecutive days (days 3–6). This drug was chosen because of its widespread clinical use and because it facilitates the extra-intestinal dissemination of *C. glabrata* despite its relatively low pathogenicity compared to *C. albicans* [54]. Non inoculated animals receiving antibiotics and immunosuppression regimen were used as internal control to assess the incidence or deaths resulting from the effect of treatments.

#### Evaluation of yeast translocation and dissemination

Four mice per *Saccharomyces* strain tested were sacrificed by cervical dislocation 5, 6 and 7 days after oral inoculation. Peyer’s patches, mesenteric lymph nodes (MLNs), livers, kidneys and brains were removed for quantitative analysis of yeast burden in each organ as described below.

### Determination of Burdens in Representative Organs and Stools

When required, stools were obtained by manually pressing the lower abdomen of mice, weighed and placed in 5 ml volumes of sterile saline solution. Mice were sacrificed by cervical dislocation and brains and kidneys or brains, kidneys and livers (depending on the assay: i.v. or oral inoculation) were obtained aseptically with forceps and scissors. In the studies of gastrointestinal infection, the intestine was then slowly draw out of peritoneal cavity with forceps and exposed on sterile gauze. The MLNs were identified and cut off. The small intestine was cut below the stomach and was first washed through with PBS using a syringe to expel the fecal matter and then thoroughly wash with PBS containing antibiotics [100 units/ml of penicillin (Sigma-Aldrich Química, S.A. Madrid, Spain), 100 µg/ml of streptomycin sulfate (Reig Jofré laboratories, S.A. Barcelona, Spain)] and an antifungal [2.5 µg/ml of amphotericin-B (Sigma-Aldrich Química, S.A.)]. Macroscopic Peyer's patches were identified and carefully dissected. All organs were kept in sterile saline solution immediately after their isolation and were later moved to a new tube with 5 ml volumes of the same solution. The organs were then homogenized with a Polytron PT 3100 instrument equipped with a 10 mm probe. Between each homogenization, the probe was cleaned and sterilized by two successive washes in non-sterile water, two successive washes with 70% ethanol and a final wash in sterile water. The number of viable yeast cells in the organs was determined by counting colony-forming units (CFU), following the plate dilution method using YPD plates with chloramphenicol (10 µg/ml) after 48 h of incubation at 30°C. All burdens have been expressed as the log_10_ geometric mean number of CFU per entire organ and per gram for stools.

Three colonies were selected randomly from each YPD plate containing each homogenized organ or stools, and were analyzed by mtDNA restriction analysis in order to confirm they belonged to the *Saccharomyces* strain inoculated.

### Statistical Analysis

To compare among strains based on the study of virulence-associated phenotypes and to analyze the association between these phenotypic traits and *in vivo* virulence by intravenous route, a presence and absence matrix was prepared from which the Spearman's Rho correlation coefficient was calculated both for strains and traits. Moreover, Jaccard proximities were calculated excluding joint absences from consideration. *P* values <0.05 were considered statistically significant (2-tailed).

In the *in vivo* studies after intravenous inoculation of *Saccharomyces* strains, burdens between brains and kidneys were compared statistically by a two-way repeated measures ANOVA (2 between-factors: strains and days; 1 within-factor: organ). Furthermore, since the Levene’s Test showed that variances were not homogenous in all cases, we performed several one-way ANOVA using the Tamhane’s Post Hoc test for multiple mean comparisons: two analyses were performed, one for each organ (brain and kidney), to study strain effect, and two more analyses were run to study the effect of time. In the *in vivo* virulence studies of oral inoculation in mice, a one-way ANOVA was used to compare burdens of isolates D14 and D23 for each organ (MLN, Peyer’s patch, liver, kidney and brain). *P* values <0.05 were considered statistically significant for all comparisons. All the statistical analyses were performed using IBM SPSS (Statistical Package for the Social Sciences) Statistics software v21 for Windows.

The Kaplan-Meier method and Wilcoxon and log-rank test were performed to compare survival curves using GraphPad software. The level of significance between curves was set at *p*<0.05.

## Results

### 1. Identification of Yeasts Isolated from Commercial Products

A total of 22 commercial products including enriched beverages and dietary products, as well as the bio-therapeutic agent sold as Ultra-Levura were microbiologically studied (see Table 1 for origin and presentation of the products). Yeast cells were isolated from only eight of them (D2, D3, D4, D6, D7, D8, D14, D23) and from Ultra-Levura (D5).

A molecular study, based on restriction analysis of the 5,8S rRNA gene and the two flanking internal transcribed sequences (ITS), was performed in six colonies randomly selected from each of the nine products to confirm their adscription to the *Saccharomyces* genus. All strains showed the typical 850 bp 5.8S-ITS PCR amplification fragment previously described for species of the *Saccharomyces sensu stricto* complex [55]. The sizes of the restriction fragments obtained with endonucleases *Cfo*I, *Hae*III and *Hin*fI (375+325+150; 325+230+170+125; 375+365+110, respectively) suggested that, as indicated on the labels, these strains belong to *S. cerevisiae* species, with the exception of D6 strain. The 5.8S-ITS fragment amplified from the latter strain showed *Cfo*I and *Hin*fI restriction patterns typical of *S. cerevisiae*, but an atypical profile was obtained when *Hae*III was used, specifically 500+230+125. According to published data, this profile may correspond to two species of the *Saccharomyces sensu stricto* complex (*Saccharomyces bayanus* and *Saccharomyces kudriavzevii*). Therefore restriction enzyme *Acc*I was used as it is able to distinguish between the two species [40]. The restriction pattern obtained (730+110) suggested that isolate D6 corresponds to the species *S. kudriavzevii* [40].

Additional restriction analyses of the nuclear genes *APM3*, *BRE5* and *PEX2* revealed that two of the isolates were in fact hybrids between *S. cerevisiae* and other *Saccharomyces* species very closely related (species belonging to the *Saccharomyces sensu strict*o complex). In the case of D6, initially identified as *S. kudriavzevii*, the restriction patterns obtained from the digestion of the *AMP3* gene with *Msp*I (750+520+310+200), digestion of the *PEX2* gene with *Hae*III (345+260+110) and digestion of the *BRE5* gene with *Hin*fI (330+289+228+153) indicated the presence of a hybrid between *S. kudriavzevii* and *S. cerevisiae*. The other isolate showing different adscription was D23; the restriction analysis of the nuclear genes *AMP3* with *Msp*I (750+680+340+280), *PEX2* with *Hae*III (345+260+180+150+110) and *BRE5* with *Hin*fI (289+228+100+66+60+43) revealed a hybrid between *S. cerevisiae* and *S. bayanus* species.

### 2. Molecular Characterization of Yeasts Isolated from Commercial Products

**Figure 1:**
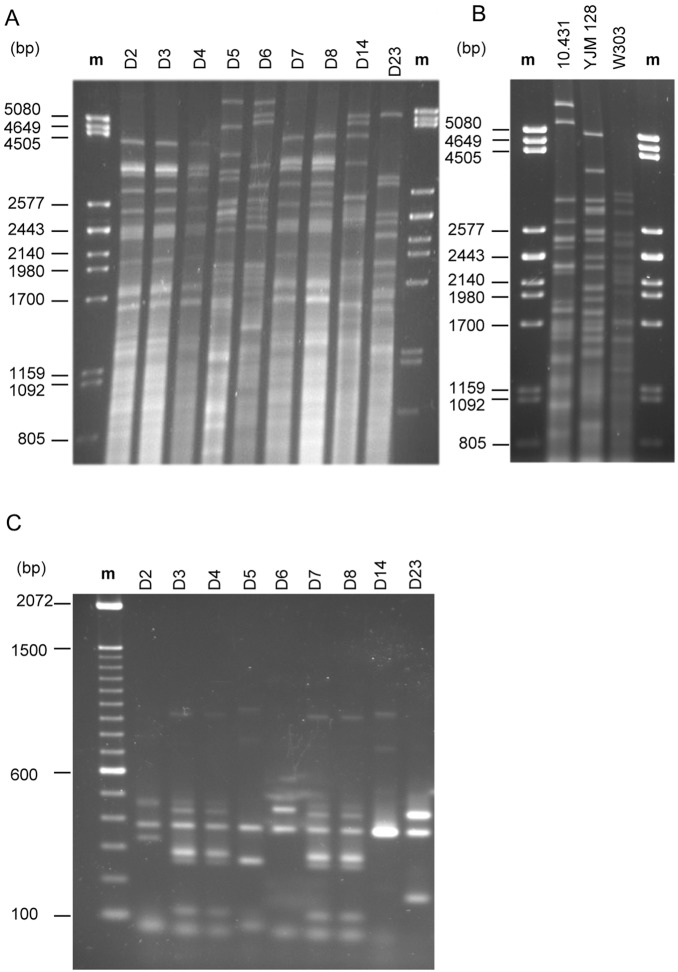
Molecular characterization of yeast strains analyzed. *Hin*fI mtDNA restriction patterns of yeasts isolated from commercial products (**A**) and of yeast strains used as controls (**B**); δ-PCR amplification patterns of yeasts isolated from commercial products (**C**). The DNA of phage λ digested with *Pst* I and a 100-bp DNA ladder marker (Invitrogen Life Technologies) served as the size standard respectively.

We studied the purity of the nine commercial products containing live yeasts by the molecular technique based on mtDNA restriction analysis with the endonuclease *Hin*fI. All six randomly selected colonies isolated from the same product showed the same restriction pattern in all cases. Moreover, strains were characterized molecularly to avoid the use of repeated strains in the *in vitro* and *in vivo* pathogenicity studies. Inter-product comparisons gave a total of five different mtDNA restriction patterns (Figure 1A). Results are summarized in Table 3 by assigning a distinct letter to each pattern differing from the others. Four restriction patterns (groups B_m_, C_m_, D_m_, E_m_) contained only one strain indicating that these strains differed from each other. One of these restriction profiles, namely B_m_, corresponded to D5 (Ultra-Levura) whose identity was confirmed by comparing its restriction profile with that obtained previously for an isolate from another commercial batch [44]. This result indicated that the bio-therapeutic strain *S. cerevisiae* var. *boulardii* was not used in the preparation of the commercial products studied here, thus we decided to include it as positive control in the following section because this strain is positive for the majority of phenotypic traits considered [28]. Only one restriction pattern (A_m_) comprised several strains (D2, D3, D4, D7, D8). Another technique, which gives good results in *S. cerevisiae* clinical strain characterization [44] is PCR amplification of δ elements. When this technique was applied, a total of six different amplification patterns were observed (Figure 1C). Results are summarized in Table 3 by assigning a distinct letter to a pattern when it differs from the others. Of the six strains forming the mtDNA group A_m_, five were identical while strain D2 grouped differently after PCR amplification. Since different levels of discrimination were obtained, the results of both techniques were combined (Table 3). As a result, six composite patterns were obtained: five corresponded to individual strains (D2, D5, D6, D14 and D23) and one was a group (A_m_/B_δ_) including four strains, three of them isolated from pills (D3, D4 and D7) and one from flakes (D8).

**Table 3:** Distribution of DNA types of yeasts isolated from commercial products.

mt DNA pattern	δ PCR pattern	Composite pattern	Product code
A_m_	A_δ_	A_m_/A_δ_	D2
A_m_	B_δ_	A_m_/B_δ_	D3, D4, D7, D8
B_m_	C_δ_	B_m/_C_δ_	D5
C_m_	D_δ_	C_m/_D_δ_	D6
D_m_	E_δ_	D_m/_E_δ_	D14
E_m_	F_δ_	E_m/_F_δ_	D23

Given that several *S. cerevisiae* strains were included as controls in the following sections, we considered it of interest to obtain their mtDNA restriction patterns in a way to assure they were clearly distinguishable from the strains under study. As shown in Figure 1B, the strains CECT 10.431, YJM128 and W303 displayed restriction patterns not found in the rest of the strains. The mtDNA restriction pattern of isolate 102 was obtained previously [44] and is different from that of the strains under study ([44]: see mtDNA pattern Y_m_ in Figure 1).

### 3. Virulence-associated Phenotypes of Yeasts Isolated from Commercial Products

Fungal properties typically found in pathogenic fungi [18]–[27] and thought to be virulence-associated traits have been studied for the dietetic strains D2, D6, D14 and D23 and one representative (D4) from the group with a composite pattern (A_m_/B_δ_).

#### 3.1. Growth at high temperatures

We analyzed the growth of the commercial yeast isolates at different temperatures (30, 37, 39 and 42°C). Strain D5 (Ultra-Levura), the clinical isolate 102, the laboratory strain W303 and the wine CECT 10.431 strains (Table 2) were also included as controls in this analysis.

**Figure 2:**
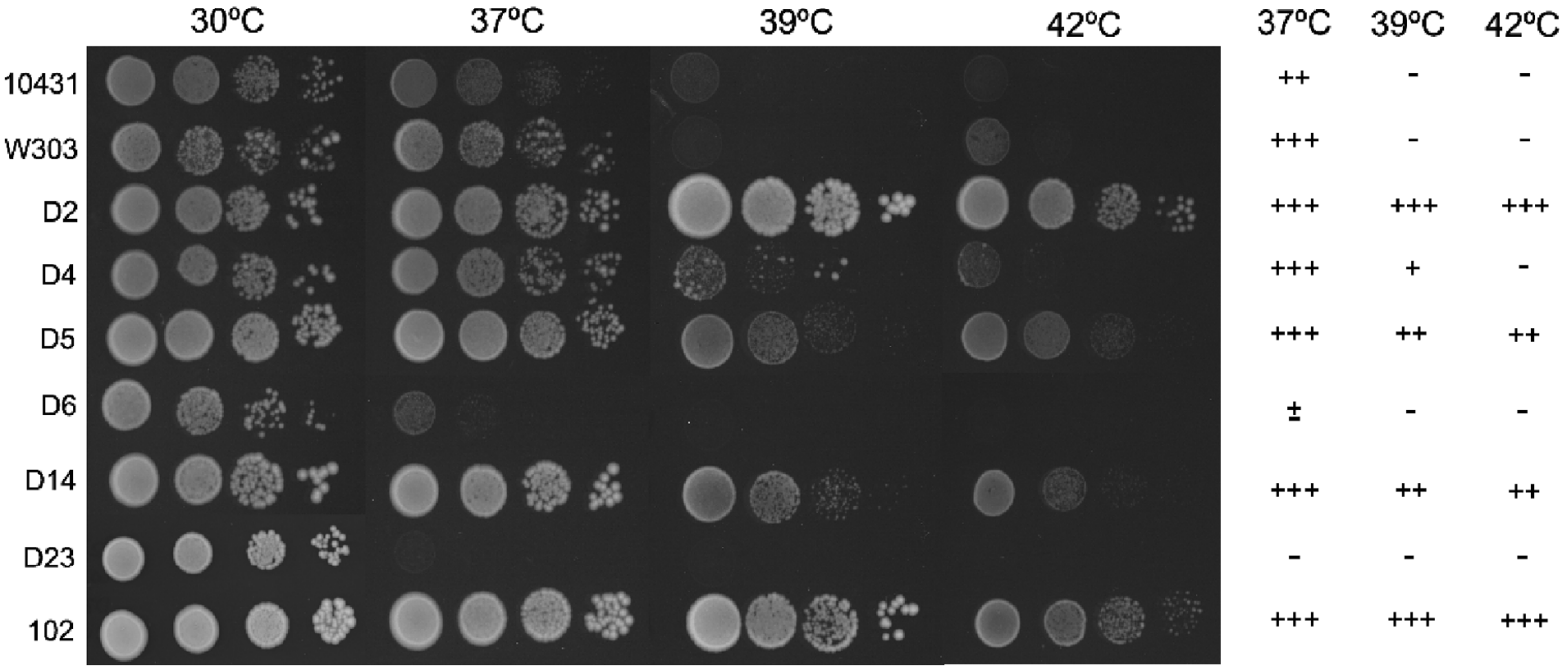
Growth at different temperatures of commercial and control *S. cerevisiae* strains. Ten-fold serial dilutions of the indicated strains were dropped on YPD plates and incubated for 24 h at 30, 37, 39 and 42°C. +++: growth in all the dilutions; ++: growth in the first two dilutions; +: growth in the first dilution; ±: growth in the first drop without dilution; –: no growth.

Figure 2 shows the growth of the commercial and control strains on YPD plates at different cell concentrations and temperatures. Strains CECT 10.431 and W303 were only able to grow up to 37°C. Meanwhile D4 strain was able to grow at 39°C albeit poorly and only D2, D5 and D14 strains grew at 42°C, as did the clinical isolate 102 whose *in vivo* virulence was previously shown to be notable since was able to cause death when tested in three murine models of i.v. inoculation [31]. Remarkably, hybrid strains (D6 and D23) only grew at 30°C. The observed growth at 42°C of strain D5 (Ultra-Levura) is in agreement with previously reported data [28].

**Figure 3:**
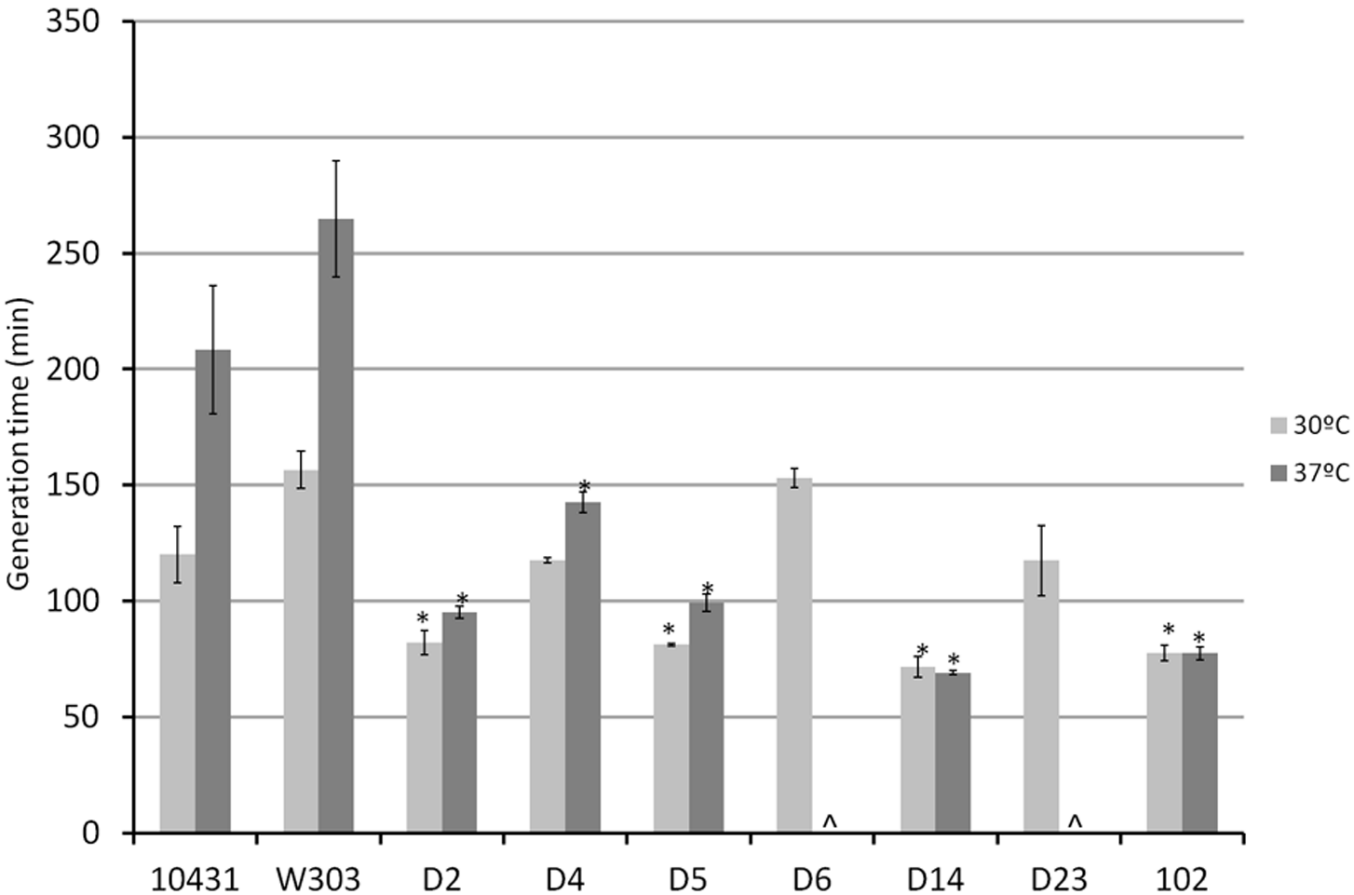
Generation time of commercial and control *S. cerevisiae* strains on YPD at 30°C and 37°C. Error bars correspond to standard deviations. ∧not determined. *p<0.05 with regard to the avirulent strains (CECT 10.431 and W303), as assessed by Students t-test.

To analyze growth at 30°C and 37°C in more detail, generation time at these temperatures was determined (Figure 3). Generation time at 37°C in strains D6 and D23 was not determined because these strains grew poorly at this temperature. Strains D2 and D14 had a significantly lower generation time at 30°C and 37°C than avirulent control strain CECT 10.431 and laboratory strain W303, but similar to that of Ultra-Levura (D5) and the clinical isolate 102. Isolate D14 showed the lowest generation times at both temperatures (about half and one third of those corresponding to control strains at 30°C and 37°C, respectively). In contrast to the other strains tested, generation time did not significantly increase at 37°C for strains D14 and 102, indicating that this high temperature is as optimum as 30°C for their growth.

#### 3.2. Extracellular secretion of degradative enzymes

**Figure 4:**
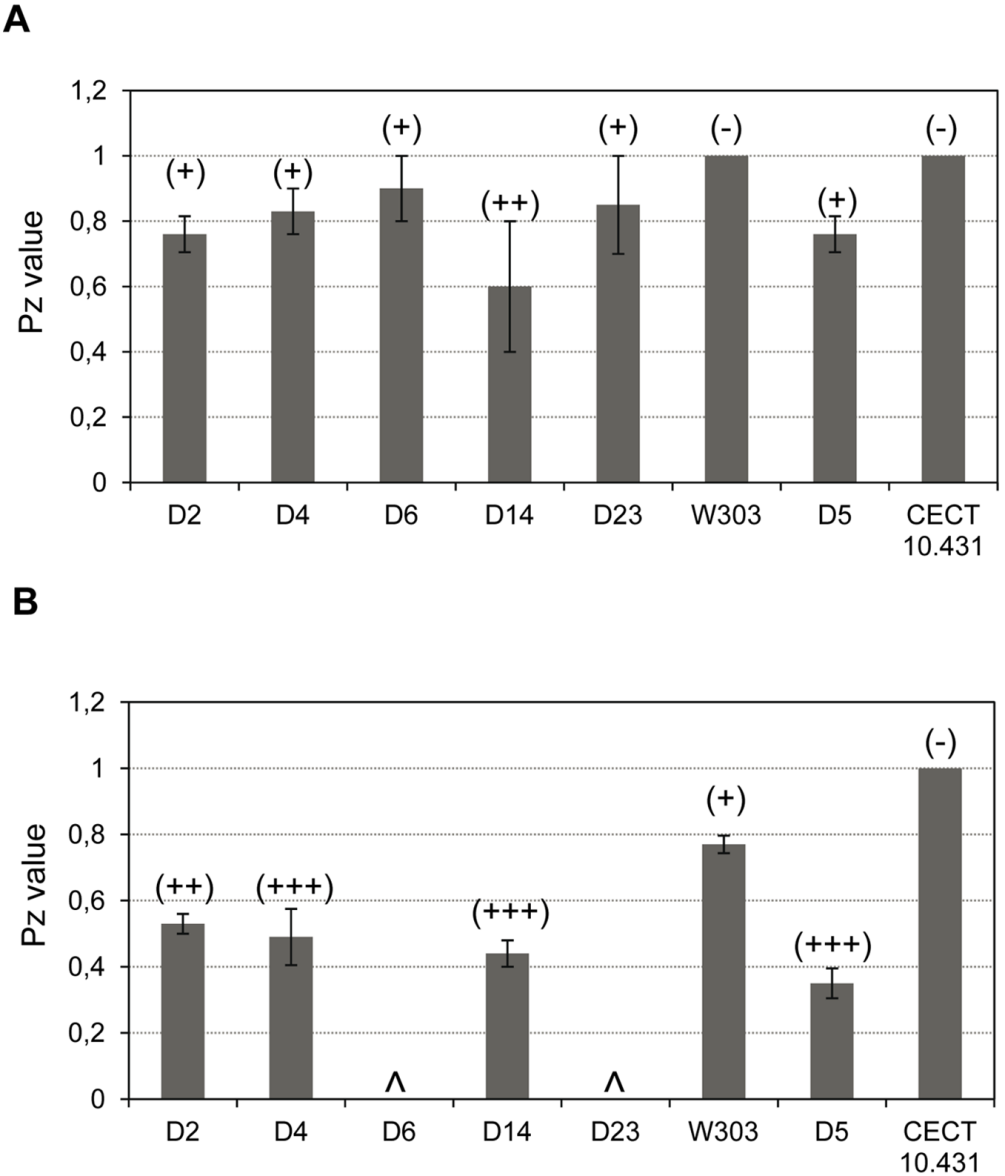
Extracellular secretion of phospholipase (A) and protease (B) of commercial and control *S. cerevisiae* strains. Activity is expressed as Pz value (see Material and Methods section) and the level of activity is indicated between brackets according the following code: Pz = 1 (Negative activity: –); 0,99≥Pz≥0,70 (Low activity: +); 0,69≥Pz≥0,50 (Moderate activity: ++); Pz≤0,50 (High activity: +++). ∧not determined (these strains were unable to growth at the optimum temperature to determine this activity). Results are expressed as the mean ± SD.

We analyzed the extracellular secretion of phospholipases and proteinases by measuring the precipitation area around each colony resulting from their activity on Egg-Yolk and BSA medium, respectively. The enzymatic activities obtained for each strain expressed as Pz values are shown in Figure 4. All isolates from dietary supplements showed phospholipase activity with levels ranging from low activity (D2, D4, D6 and D23) to moderate activity (D14). Laboratory strain W303 and wine strain CECT 10.431 showed no activity while the D5 strain secreted the enzyme but at low level.

Protease activity could not be assessed for the hybrid isolates (D6 and D23) because, as mentioned above, both isolates were unable to grow at 37°C (optimum temperature to determine this activity). While D4 and D14 showed high activity, as did strain D5 (Ultra-Levura) used as positive control [28], D2 displayed moderate activity levels (Pz = 0.53) showing an intermediate level of activity between the positive (D5) and negative (CECT 10.431) controls. The laboratory strain W303 showed a high Pz value (0.77) corresponding to low activity.

#### 3.3. Pseudohyphal formation

**Figure 5:**
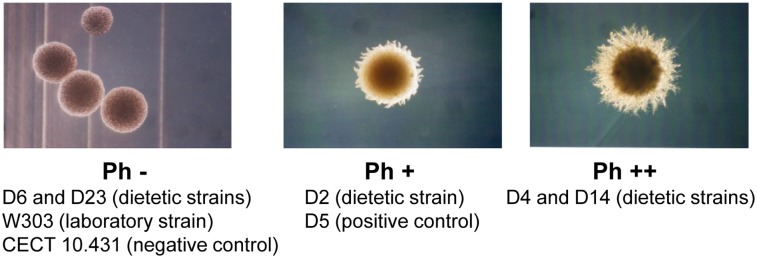
Differences in pseudohyphal growth of commercial and control *S. cerevisiae* strains in SLAD medium. Examples of colony morphologies of pseudohyphal growth: (Ph −) absence of pseudohyphal growth, (Ph +) intermediate pseudohyphal development, (Ph ++) high pseudohyphal development. The images were taken with a Leica Camera associated with a microscope Leica HPS60 at 40X magnification.

We analyzed pseudohyphal growth and found different degrees of pseudofilamentation in the assayed strains. We considered the absence or presence of this type of growth, morphology and the length of the pseudohyphal chains (Figure 5). Isolate D2 showed intermediate pseudohyphal growth (Ph +), as did control strain D5 [28], while isolates D4 and D14 exhibited high pseudohyphal development (Ph ++) based on the number and length of filaments. The hybrid isolates (D6 and D23) and the laboratory strain W303 showed an absence of pseudohyphal growth (Ph −), behaving the same as the negative control (CECT 10.431).

#### 3.4. Invasive growth

Some *S. cerevisiae* isolates are capable of penetrating agar, producing filaments able to resist vigorous washing of the surface of solid growth medium. This feature is known as invasive growth [56].

**Figure 6:**
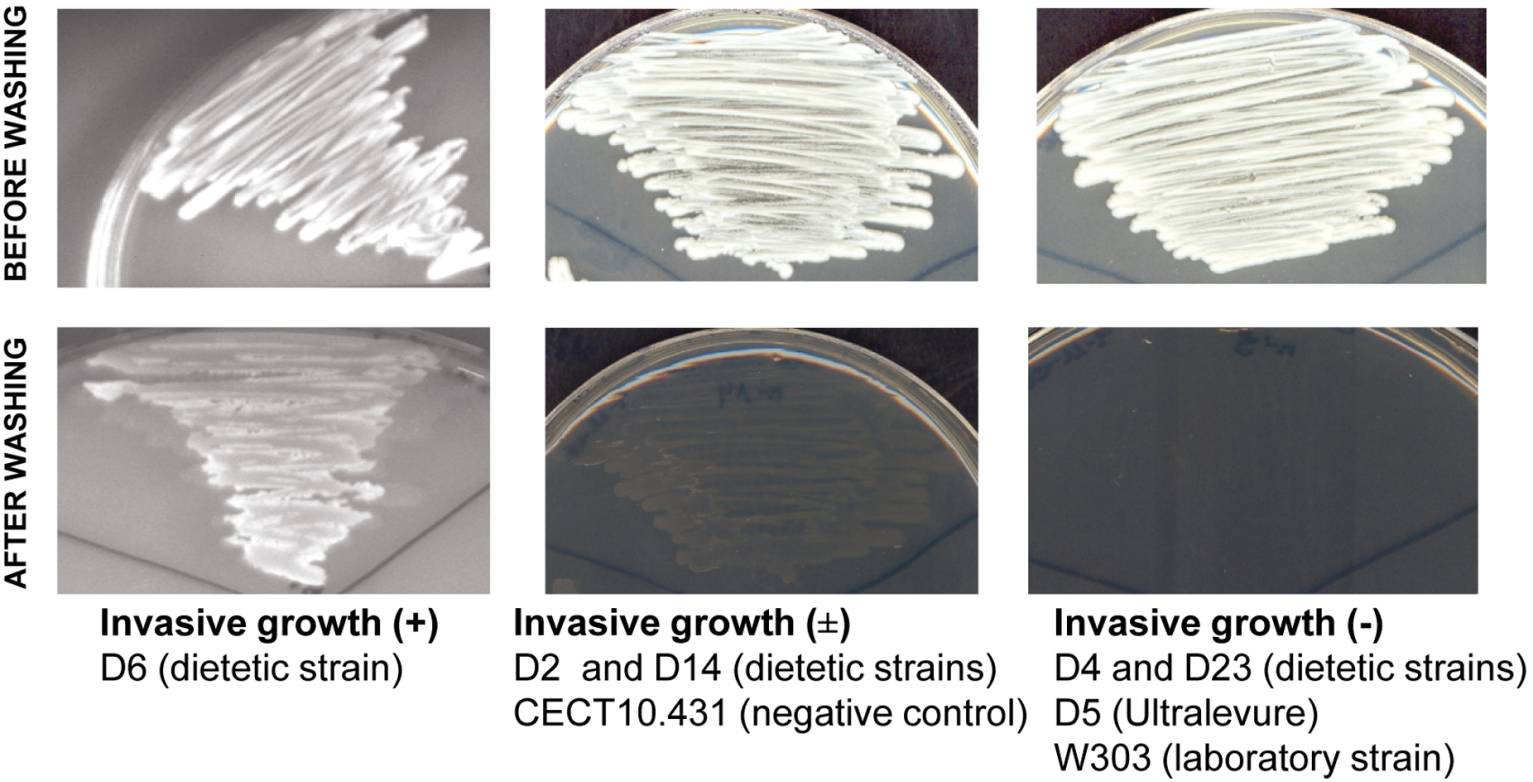
Examples of invasive growth in commercial and control *S. cerevisiae* strains. Three degrees of invasiveness were determined based on the residual growth remained after washing. Invasive growth (+): yeast cells attached to the agar surface; Invasive growth (±): yeast cells partially anchored to the agar; Invasive growth (–): yeast cells fully eliminated.

Three different behaviors regarding invasive growth were observed in the assayed strains, based on the residual growth left after washing the cultures grown on YPD plates for five days at 30°C (Figure 6). Isolates D4 and D23, together with the laboratory strain W303 and the control strain D5, were fully eliminated after washing (negative invasive growth: –). D2 and D14 remained partially anchored to the agar, forming a thin layer of yeast (intermediate invasive growth: ±); this type of growth was also shown by the control strain CECT 10.431. Only isolate D6 showed positive invasive growth (+), with practically all the culture being attached to the agar surface after washing.

#### 3.5. Colony phenotype switching

The switching phenomenon, described in some fungi like *C. albicans*, *C. tropicalis* or *C. neoformans*, is a mechanism of phenotypic plasticity manifested by spontaneous reversible changes in colony morphology occurring at a frequency of about 10^−3^–10^−4^. We studied this phenomenon in four of the strains isolated from dietary supplements (D2, D4, D14, and D23) by analyzing the phenotypic changes in a number of colonies between 6000 and 8000. All four strains showed a main phenotype and several secondary phenotypes; the latter with a frequency of appearance between 3.4×10^−2^ and 1.91×10^−4^ (Table 4). Specifically nine colony phenotypes were observed in D2 strain, six in D4 strain and eight in strains D14 and D23. An example of the colony phenotypes obtained for strain D2 and D14 is shown in Figure S1. Stability and reversion of some of these secondary phenotypes were also studied. There were reversible and stable secondary phenotypes in the four strains analyzed (data not shown). The stable secondary phenotypes were able to revert to the main phenotype at low frequency, except two secondary phenotypes of strains D4 and D14. These results indicate that these strains showed colony switching following a similar pattern.

**Table 4:** Colony phenotype switching frequencies of commercial *Saccharomyces* strains.

	Principal phenotype	Secondary phenotypes							
Strain	Colony number (frequency)	Colony number (frequency)							
D2	**D2-A**	**D2-B**	**D2-C**	**D2-D**	**D2-E**	**D2-F**	**D2-G**	**D2-H**	**D2-I**
	10.332 (0.99)	71 (6.78×10^−3^)	31 (2.96×10^−3^)	10 (9.56×10^−4^)	3 (2.87×10^−4^)	7 (6.69×10^−4^)	2 (1.91×10^−4^)	2 (1.91×10^−4^)	2 (1.91×10^−4^)
D4	**D4-A**	**D4-B**	**D4-C**	**D4-D**	**D4-E**	**D4-F**			
	7715 (0.95)	65 (7.98×10^−3^)	284 (3.48×10^−2^)	7 (8.59×10^−4^)	6 (7.36×10^−4^)	73 (8.96×10^−3^)			
D14	**D14-A**	**D14-B**	**D14-C**	**D14-D**	**D14-E**	**D14-F**	**D14-G**	**D14-H**	
	6565 (0.99)	18 (2.27×10^−3^)	4 (6.06×10^−4^)	2 (3.03×10^−4^)	3 (4.54×10^−4^)	6 (9.09×10^−4^)	2 (3.03×10^−4^)	1 (1.51×10^−4^)	
D23	**D23-A**	**D23-B**	**D23-C**	**D23-D**	**D23-E**	**D23-F**	**D23-G**	**D23H**	
	8284 (0.96)	287 (3.32×10^−2^)	13 (1.50×10^−3^)	19 (2.20×10^−3^)	2 (2.31×10^−4^)	12 (1.39×10^−3^)	23 (2.66×10^−3^)	2 (2.31×10^−4^)	

Colony phenotype descriptions: D2-A (5–7 mm, light pink, strong pink centre, white edge, creamy texture); D2-B (5–7 mm, light pink, strong pink centre, white edge, strong pink sector, creamy texture); D2-C (5–7 mm, strong pink, white edge, rough texture, striated); D2-D (3 mm, light pink, strong pink centre, creamy texture); D2-E (5–7 mm, light pink, strong pink centre, white edge, creamy texture, star shape); D2-F (5–7 mm, light pink, strong pink centre, white edge, white sector, creamy texture); D2-G (3 mm, strong pink, creamy texture, irregular); D2-H (4 mm, light pink, strong pink centre, white edge, strong pink sector, rough texture); D2-I (5–7 mm, light pink, strong pink centre, rough texture, striated); D4-A (5 mm, light pink, strong pink centre, creamy texture); D4-B (2 mm, light pink, strong pink centre, creamy texture, irregular); D4-C (1 mm, light pink, creamy texture, irregular); D4-D (3 mm, strong pink, creamy texture); D4-E (5 mm, light pink, strong pink centre, creamy texture, white sector); D4-F (5 mm, light pink, strong pink centre, creamy texture, irregular); D14-A (5 mm, light pink, pale pink centre, strong pink edge, creamy texture); D14-B (2–3 mm, pale pink, creamy texture); D14-C (2–3 mm, light pink, pale pink centre, creamy texture); D14-D (5 mm, light pink, pale pink centre, strong pink edge, creamy texture, white sector); D14-E (5 mm, light pink, pale pink centre, strong pink edge, creamy texture, strong pink sector); D14-F (5 mm, medium pink, pale pink centre, strong pink edge, creamy texture); D14-G (5 mm, light pink, pale pink centre, strong pink edge, creamy texture, striated); D14-H (2 mm, pale pink, dark pink edge, creamy texture); D23-A (4–5 mm, light pink, strong pink centre, creamy texture); D23-B (1–2 mm, strong pink, creamy texture); D23-C (4–5 mm, light pink, strong pink centre, creamy texture, irregular); D23-D (4 mm, strong pink, creamy texture); D23-E (7–8 mm, light pink, strong pink centre, creamy texture); D23-F (4–5 mm, light pink, strong pink centre, rough texture, striated); D23-G (5 mm, light pink, strong pink centre, strong pink sector, creamy texture); D23-H (1–2 mm, light pink, strong pink centre, creamy texture).

#### 3.6. Activation of MAPK signaling pathways

**Figure 7:**
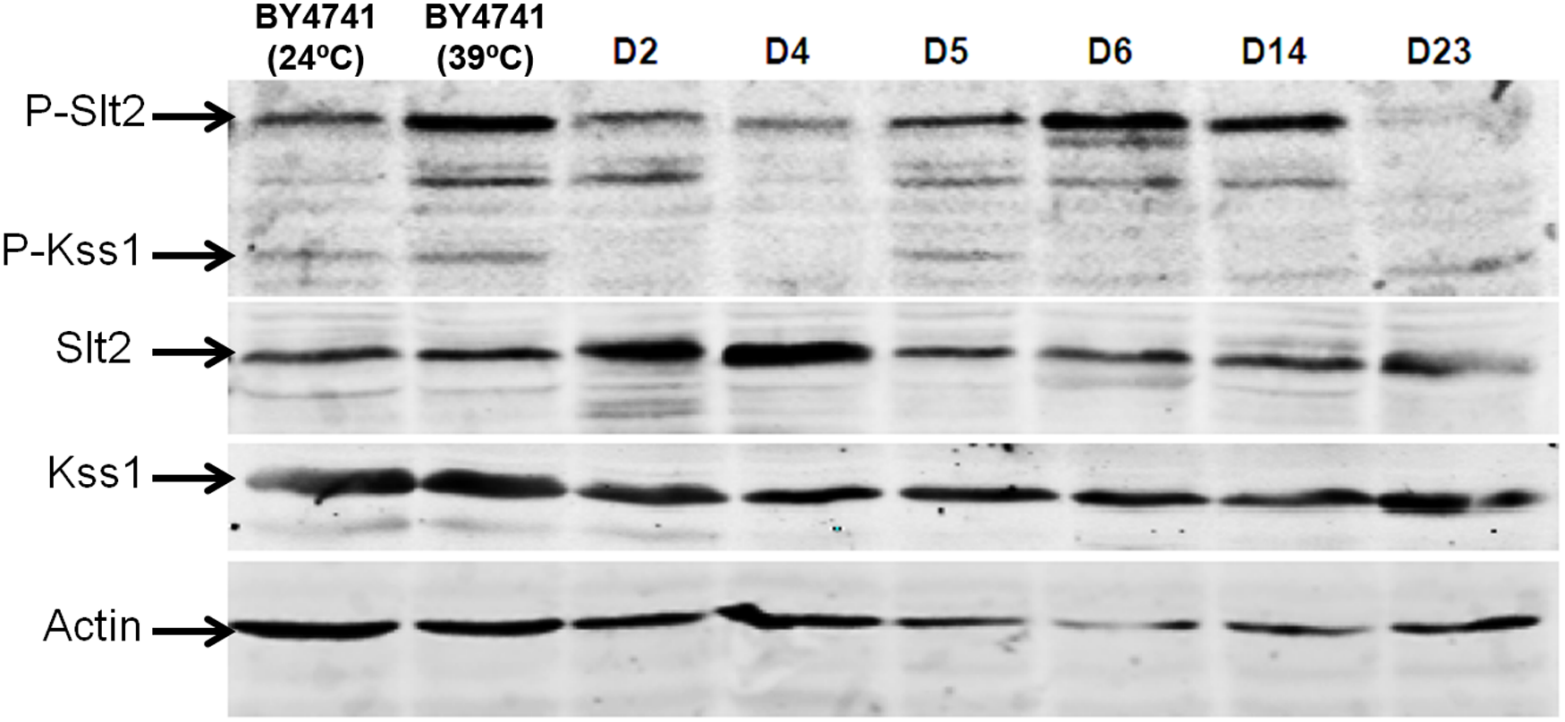
Differences in phosphorylation of MAPKs Slt2 and Kss1 in dietary *Sacharomyces* strains. Phosphorylation of MAPKs Slt2 and Kss1 in extracts from the indicated commercial *S. cerevisiae* strains growing at 24°C and from the laboratory BY4741 strain, growing both at 24°C and 39°C. Phospho-Kss1 and phospho-Slt2 were detected by immunoblotting analysis with anti-phospho-p42/44 and the protein load monitored using anti-Slt2, anti-Kss1 and anti-actin antibodies.

To study the basal level of activation of the pathways mediated by Slt2 and Kss1 in dietary and probiotic *Saccharomyces* strains, the dually phosphorylated forms of these proteins were detected by immunoblotting with polyclonal anti-phospho-p44/p42 MAPK (Thr-202/Tyr-204) antibody that specifically recognizes activated MAPKs [51]. Anti-Slt2 and anti-Kss1 antibodies were used to detect the total amount of these proteins. Western blotting was performed with extracts of the different strains grown at 24°C, in the absence of external stimulating conditions of these MAPKs signaling pathways. Extracts of the laboratory strain BY4741 grown at 24°C and 39°C were used as controls respectively for basal phosphorylation of these MAPKs and activation of Slt2 upon stimulation of the CWI pathway, as in a previous work [30]. Differences in phosphorylation of Slt2 and Kss1 were observed among the different strains tested (Figure 7). Greater phosphorylation of Slt2 was observed in strains D5, D6 and D14 as compared to control laboratory strain under the same conditions. An increased level of phospho-Kss1 was only detected in strain D5 (Ultra-Levura), in agreement with a previous study [30].

#### 3.7. Adherence to polystyrene plastic

**Figure 8:**
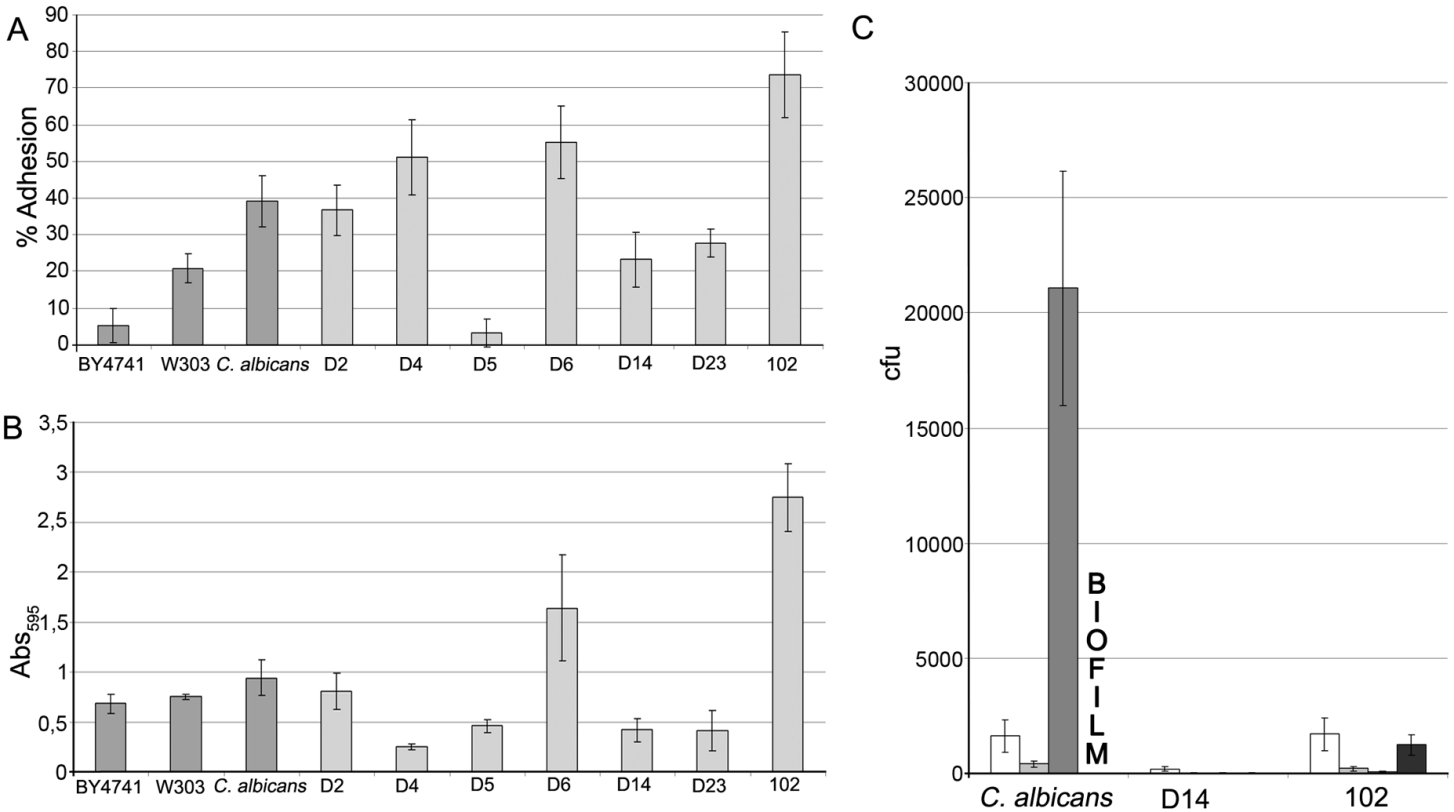
Adherence of commercial and control yeast strains to plastic and catheters. (A) Percentage of cells of the different yeast strains adhered to polystyrene Petri plates incubated 1 h at 37°C with 5% CO_2_ in glucosaline solution. (B) Adherence of yeast strains to polystyrene microtiter plates incubated 1 h at 37°C with 5% CO_2_ in glucosaline solution determined by absorbance. (C) Adherence of yeast strains to sections (1 cm) of polyurethane intravenous catheters incubated 1 and 24 h at 37°C in glucosaline solution and RPMI. First bar: 1 h, glucosaline solution; Second bar: 24 h, glucosaline solution; Third bar: 1 h, RPMI medium; Fourth bar: 24 h, RPMI medium).

In *Candida spp.* adherence to host tissues and plastic surfaces has been associated with pathogenesis, because it is the first step in colonization and development of infection [25], [26]. For this reason, we analyzed the adherence of the *Saccharomyces* strains under study to different plastics. To test adherence to polystyrene, two materials were used: petri plates and 96-well microtiter plates. Laboratory strains BY4741 and W303 were used as negative controls and strain SC5314 of *C. albicans* was chosen as positive control for adherence. We also tested the *S. cerevisiae* clinical isolate 102. As shown in Figure 8, strain D5 showed very low adherence to Petri plates (even lower than control strain W303), whereas strains D2, D4 and D6 showed similar adhesion to those displayed by the clinical strain 102 and the *C. albicans* strain SC5314. Strains D14 and D23 showed lower adhesion rates, similar to that of laboratory strain W303 and greater than that of laboratory strain BY4741 (Figure 8). In the adherence assay on 96-well microtiter plates strain D6 showed the greatest adhesion among the dietary and probiotic strains while the other strains gave values equal to or lower than the laboratory control strains. Remarkably, whereas strain 102 displayed high adherence in this assay, the *C. albicans* strain did not show greater adherence than laboratory strains of *S. cerevisiae*.

In summary, high adherence to polystyrene was observed for strains D6 and 102 with both methods, while strain D5 showed the lowest adhesion rates.

#### 3.8. Adherence to polyurethane intravenous catheters

Intravenous catheters constitute a route of entryof microorganisms into the bloodstream. Thus, we decided to test the adherence of the D14 strain to intravenous catheters because its virulence in murine model has been described [37] and the 102 clinical strain due to its observed high adhesion to polystyrene. We also used the SC5314 strain of *C. albicans* as a positive control of adhesion. These experiments were performed on polyurethane catheters used for critically ill patients (long-term catheterized). Glucosamine solution was used because this medium mimics the environment in contact with the catheter lumen and cell culture medium RPMI because it mimics the contact area with the external side of the catheter.

As shown in Figure 8C, the strain D14 showed low adherence in both experiments. In contrast, the clinical *S. cerevisiae* isolate 102 and the *C. albicans* SC5314 strain displayed similar high adherence to polyurethane in glucosamine solution, which is consistent with their ability to adhere to polystyrene, although the adherence of the 102 strain was lower than that of *C. albicans* when RPMI is used. After 24 h in such medium, *C. albicans* was the only strain able to form a biofilm adhered to catheters. For this reason it was not possible to quantify the number of adhered cells, although biofilm formation implies high adhesion ability.

### 4. *In vivo* Virulence by Intravenous Inoculation in Mice

The results of the analysis of virulence-associated phenotypes (summarized in Table 5) show that isolates D2, D4 and D14 displayed positivity for 63.6% to 72.7% of the phenotypic traits studied (Table 5). Specifically, the three isolates were able to grow at 37°C and 39°C, showed pseudohyphal growth and secreted proteases and phospholipases. The only difference was the ability to grow at 42°C which was negative for D4 and activation of MAPK Slt2 that was positive only for D14. The association perceived between D2, D4 and D14 based on their *in vitro* behavior was shown positive and statistically significant in terms of the Spearman’s correlation coefficient (*p* = 0.01 between D2 and D4; *p* = 0.030 between D2 and D14) and this result is consistent with the analysis performed using the Jaccard Proximities test that displayed the maximum similarity values (between 0.700 and 0.889) for these three strains. Furthermore, D2, D4 and D14 were clearly differentiated from the two hybrid strains (D6 and D23) (*p*>0.05, Spearman’s correlation coefficient; similarity values between 0.182–0.250, Jaccard Proximities test). Taking into account that these results indicated that the group of isolates named D2, D4 and D14 could be potentially virulent, we decided to study their virulence *in vivo*. Moreover, we considered of interest to determine if there was association between absence of virulence-associated phenotypes and pathogenicity *in vivo*. To do so, we also tested D23 as it was the isolate with the lowest percentage of positive traits (20%) compared to D6 (44.4%).

**Table 5:** Virulence-associated phenotypes observed in strains isolated from commercial products.

Strain	Growth at:			Enzymatic activity		Growth		Switching	MAPK activation		Plastics ADH	% Positivity
	37°C	39°C	42°C	Protease	PLA	Ph	Invasive		Slt2	Kss1		
D2	+	+	+	+	+	+	−	+	−	−	+	72.7
D4	+	+	−	+	+	+	−	+	−	−	+	63.6
D14	+	+	+	+	+	+	−	+	+	−	−	72.7
D6	−	−	−	n.d.	+	−	+	n.d.	+	−	+	44.4
D23	−	−	−	n.d.	+	−	−	+	−	−	−	20.0

Phenotype traits shown in Figures (2, 4, 5, 6, 7, 8) and Table 4 were simplified as follows to calculate the Spearman’s Rho correlation coefficients between strains and apply the Jaccard proximity test (excluding joint absences for considerations): growth at 37, 39 and 42°C, +++, ++ and + were simplified to +, and ± and − were simplified to −; phospholipase activity (PLA), ++ and + were simplified to +; pseudohyphal (Ph) growth,++ and + were simplified to +; invasive growth, ± and − were simplified to −; switching, multiple colony phenotypes with frequencies between 10^−2^−10^−4^ were considered +; MAPK activation, high Slt2 and Kss1 phosphorylation was considered + and low or non phosphorylation were considered −; Plastics adherence (ADH), Petri plates adherence values >30% and microtiter plates absorbance values >1 were considered +, Petri plates adherence values ≤30% and microtiter plates absorbance values <1 were considered −. n.d. not determined.

We evaluated the potential ability of D2, D4, D14 and D23 to develop systemic infection after their intravenous inoculation (i.v.) in immunocompetent BALB/c mice and we compared the results with those of the laboratory strain W303, the wine strain CECT 10.431 and the clinical strain YJM128. The strains CECT 10.431 and YJM128 were chosen as non-pathogenic and pathogenic controls because they displayed low and high capacity to lodge and proliferate in representative organs respectively, after intravenous inoculation of mice [16], [31], [37].

**Figure 9:**
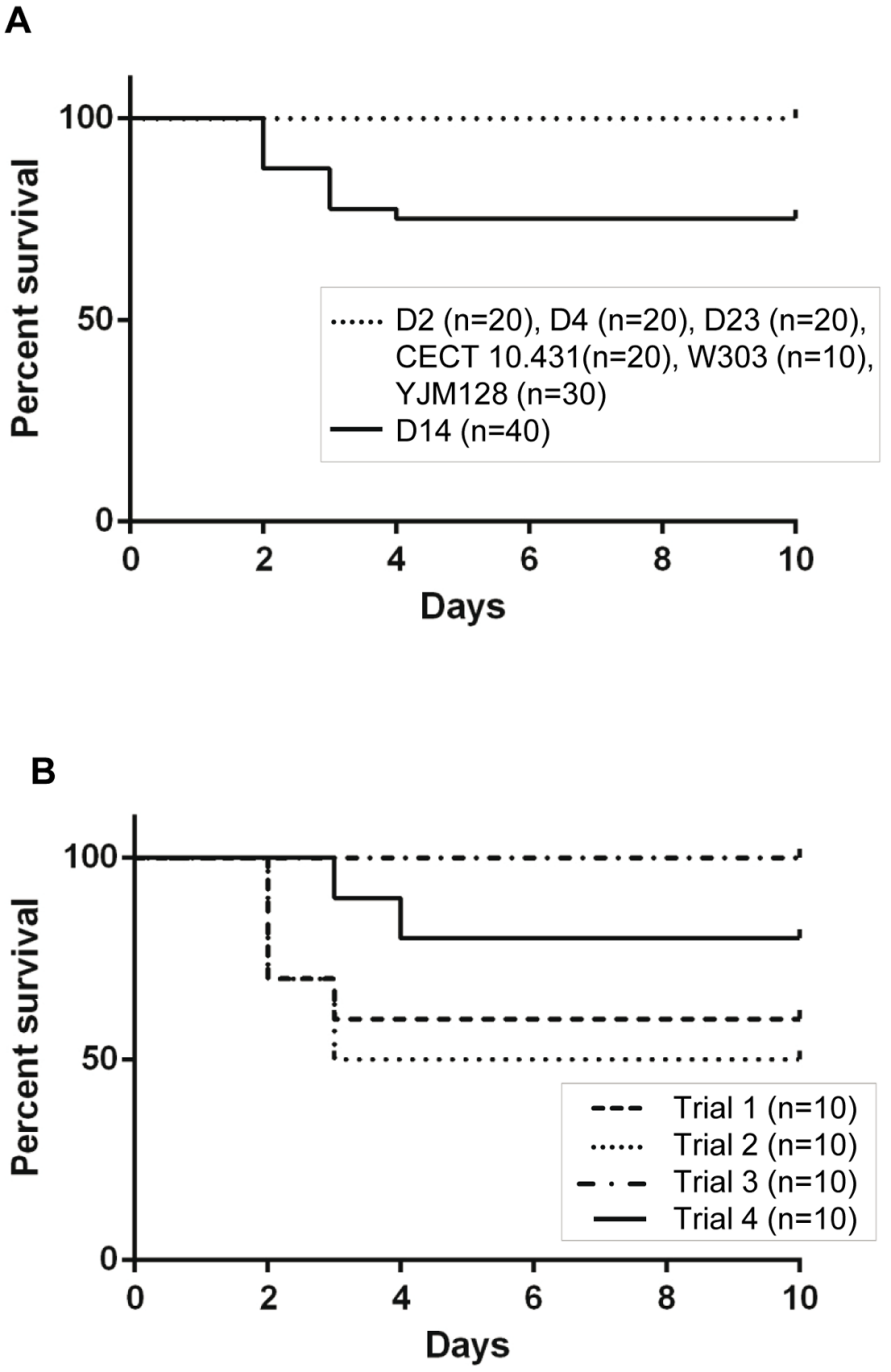
Kaplan-Meier plot of the cumulative mortality of BALB/c mice intravenously challenged with *Saccharomyces* strains. (A) D14 survival curve representing the combination of four experiments was compared to that of the rest of dietetic strains (D2, D4, D23) and control strains (CECT 10.431, W303, YJM128); (B) Comparison of D14 survival curves obtained in four independent trials.

Pathogenicity was evaluated according to death rates and by determining colonization ability (the presence of yeast cells in brains and kidneys as demonstrated by culture). Three days after intravenous inoculation, mice inoculated with strains D2, D4, D14, and the virulent control YJM128 strain showed signs of infection, in particular, weight loss, hair frizz and low mobility, which could indicate a febrile process. After the first seven days, most of affected mice recovered, except an average of 27.5% of those inoculated with isolate D14, which died between the second and fourth days post-infection (Figure 9A). The difference of mortality observed between mice inoculated with D14 and the rest of isolates was statistically significant (*p*<0.05, log rank and Wilcoxon tests). Therefore, D14 was the only commercial isolate causing lethality among the assayed strains. As observed in Figure 9B, the number of dead mice due to i.v. inoculation of D14 varied depending on the assay. The differences in mortality rates found between the four trials were equivalent (*p*>0.05, log rank and Wilcoxon tests).

**Figure 10:**
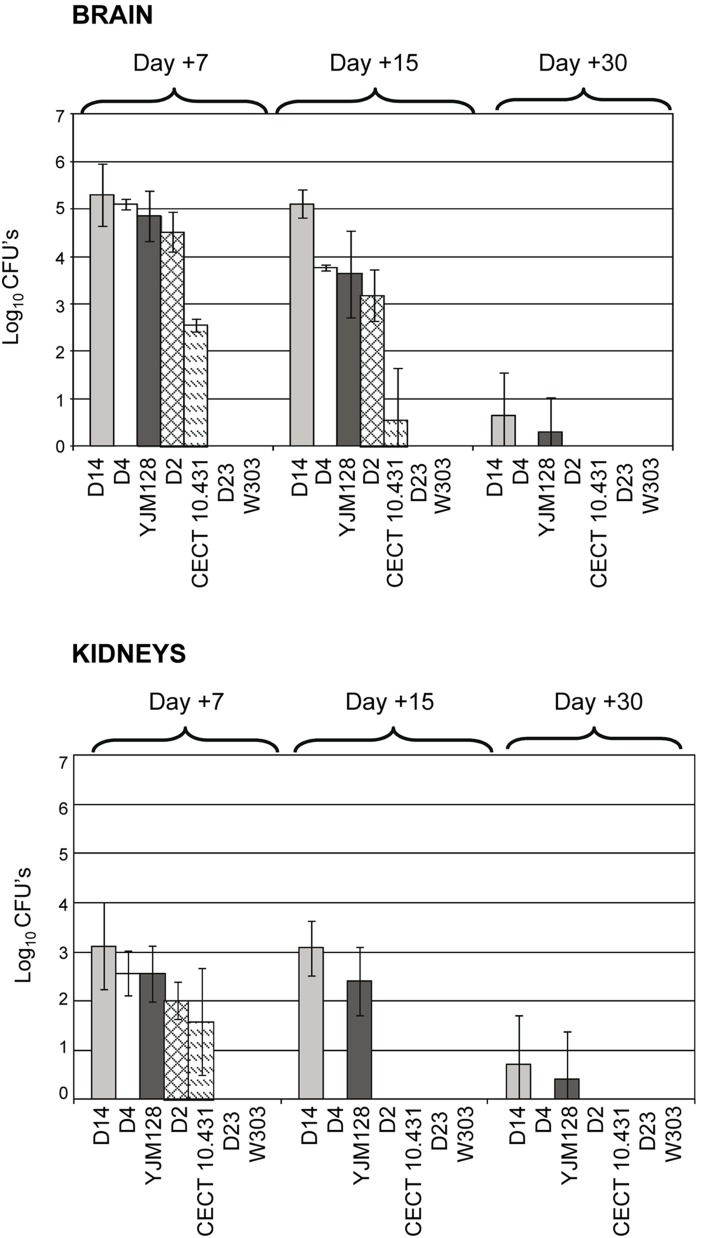
Comparative burdens of *Saccharomyces* strains recovered in brains and kidneys after intravenous infection of BALB/c mice. Day +7: D2, D4, W303 and CECT 10.431 (*n* = 4 each); D14 and D23 (*n* = 6 each); YJM118 (*n* = 10). Day +15: W303 (*n* = 3); D2, D4 and CECT 10.431 (*n* = 4 each); D14 and D23 (*n* = 7 each); YJM118 (*n* = 9). Day +30: W303 (*n* = 3); D2, D4, D23 and CECT 10.431 (*n* = 6 each); D14 and YJM128 (*n* = 11). Results are expressed as the mean ± SD.

Virulence was also evaluated by determining the burden of each strain in brains and kidneys during the 30-day experiment. As shown in Figure 10, all isolates of dietary products, except strain D23, were able to colonize the brains and kidneys of BALB/c mice. It is worth noting that D23 and the laboratory strain (W303), which also exhibited undetectable loads in these organs, were negative for most of the phenotypic traits associated with virulence analyzed. Moreover, all strains showed a clear preference for the brain, since the burdens detected in this organ were significantly higher than those recorded in kidneys (*p*<0.05, two-way repeated measures ANOVA analysis). The differences between the two organs were especially evident at day 15 post-infection, since some isolates (D2, D4 and CECT 10.431) were detectable in brain, but not in kidneys.

When strains were compared regarding burden data obtained in brains at day +7, two groups of strains were observed: D2, D4 and D14 that presentedburden levels equivalent to those of the positive control (YJM128) (*p*>0.05, one-way ANOVA followed by Tahmahe’s post-test) and higher than those of the negative control (CECT 10.431), although this difference was not statistically significant (*p*>0.05, one-way ANOVA analysis followed by Tahmahe’s post-test. In contrast, D23 and the laboratory strain W303, were totally undetectable in these organs at any time (Figure 10). In kidneys at day +7, we observed the same behavior as in brains, differences in burdens levels were appreciable but were not statistically significant (*p*>0.05, one-way ANOVA followed by Tahmahe’s post-test).

The differences between isolates became evident at day 15 post-infection. Therefore, burdens at this time provide information about the clearance profile of each strain and hence their pathogenic potential. As shown in Figure 10, the burden values in brains at this time were significantly lower than those obtained on day 7 in this organ for all strains (*p*<0.05, one-way ANOVA followed by Tahmahe’s post-test) except for D14, indicating that D14 is the one with the greatest resistance to clearance. D14 is also remarkable because it shows significantly higher burden levels than the other isolates, including the positive control (YJM128) (*p*<0.05, one-way ANOVA followed by Tahmahe’s post-test). Moreover, burden values shown by D2 and D4 in brains were equivalent to those shown by the positive control (YJM128) (*p*>0.05, one-way ANOVA followed by Tahmahe’s post-test), and clearly higher than those of the negative control (CECT 10.431) (*p*<0.05, one-way ANOVA followed by Tahmahe’s post-test). However, at day 15 post-infection, kidney clearance was particularly evident in D2 and D4, as both isolates showed undetectable burdens as did the negative control. Again, D14 stood out since it was the only dietetic isolate able to persist in this organ and at levels equivalent to those shown at day 7 (*p*>0.05, one-way ANOVA followed by Tahmahe’s post-test). This pattern of behavior is comparable to the strain used as positive control (YJM128). By day 30 post-infection, the majority of isolates had been completely eliminated in both organs except strains D14 and YJM128, which were detected at statistically insignificant levels compared to the negative control (*p*>0.05, one-way ANOVA followed by Tahmahe’s post-test).

In summary, according to the degrees of virulence proposed by Byron *et al*. [15] based on burden levels and ability to cause death, D14 could be considered a virulent isolate; D2 and D4 isolates, despite showing high burden levels in brains at 7 and 15 days post-infection, were unable to cause death and, therefore, can be considered of low virulence; D23 together with laboratory strain W303 can be considered avirulent, given their inability to colonize BALB/c mice after intravenous inoculation.

### 5. *In vivo* Virulence by Oral Inoculation in Mice

The *in vivo* virulence study by i.v. inoculation described in the previous section has shown the potential pathogenicity of some *Saccharomyces* strains isolated from dietary products. It should be noted that due to the oral entry of yeast cells contained in such preparations, only those strains capable of passing through the gastrointestinal barrier and spreading are potentially dangerous. According to death rates and burden data obtained by i.v. inoculation, D14 is the isolate posing the greatest risk. Therefore, its infectivity was assessed in an experimental murine model of gastrointestinal infection with immunosupression and disruption of mucosal integrity, and compared with the avirulent D23 isolate.

**Figure 11:**
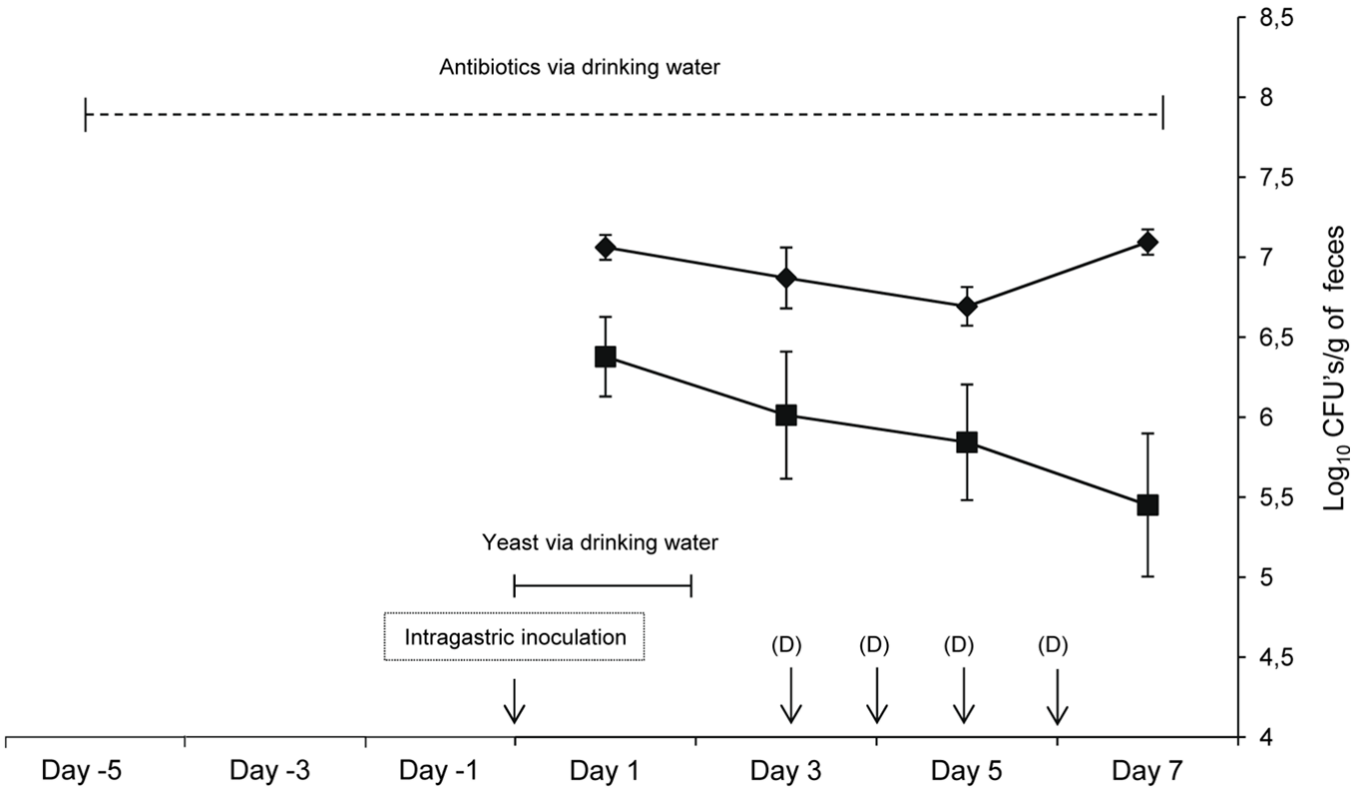
Evaluation of the presence of yeasts in mouse feces during the assay of gastrointestinal infection. Fecal counts of dietetic strains D14 (♦) and D23 (▪) after oral administration of ICR/Swiss mice receiving antibiotic supplementation in the drinking water and immunosuppressant intraperitoneally are shown. Each point represents the mean of 12 mice (day 1), 6 mice (day 3), 5 mice (day 5) and 4 mice (day 7). Results are expressed as the mean ± SD. (D): Dexamethasone injected intraperitoneally.

The presence of yeasts in the intestinal tract was evaluated by determining fecal load from the beginning of administration (day 0) to the end of the trial (day 7) (Figure 11). One day after oral challenge, stool cultures from all mice tested positive for both strains. Yeast levels remained at values between 6.5 and 7.3 log_10_ CFU/gram of feces for D14 and between 4.9 and 6.8 log_10_ CFU/gram for D23, during the entire experiment even after stopping the administration of the corresponding strain in drinking water (from day 3). According to these data, the treatment with an antibiotic regimen kept D14 and D23 in the gastrointestinal tract at levels in the same order as those achieved by prior researchers for other yeast species [57], [58] and, therefore, they were sufficient to undertake the study of intestinal infection.

Antibiotic administration was maintained throughout the experiment to minimize complications of bacterial infections occurring as a result of dexamethasone treatment. Although the use of antibiotics did not completely eliminate the bacterial flora (data not shown), residual bacteria did not have a significant pathogenic effect, as indicated by the fact that control mice that were not inoculated with yeast cells neither showed signs of infection nor died.

**Figure 12:**
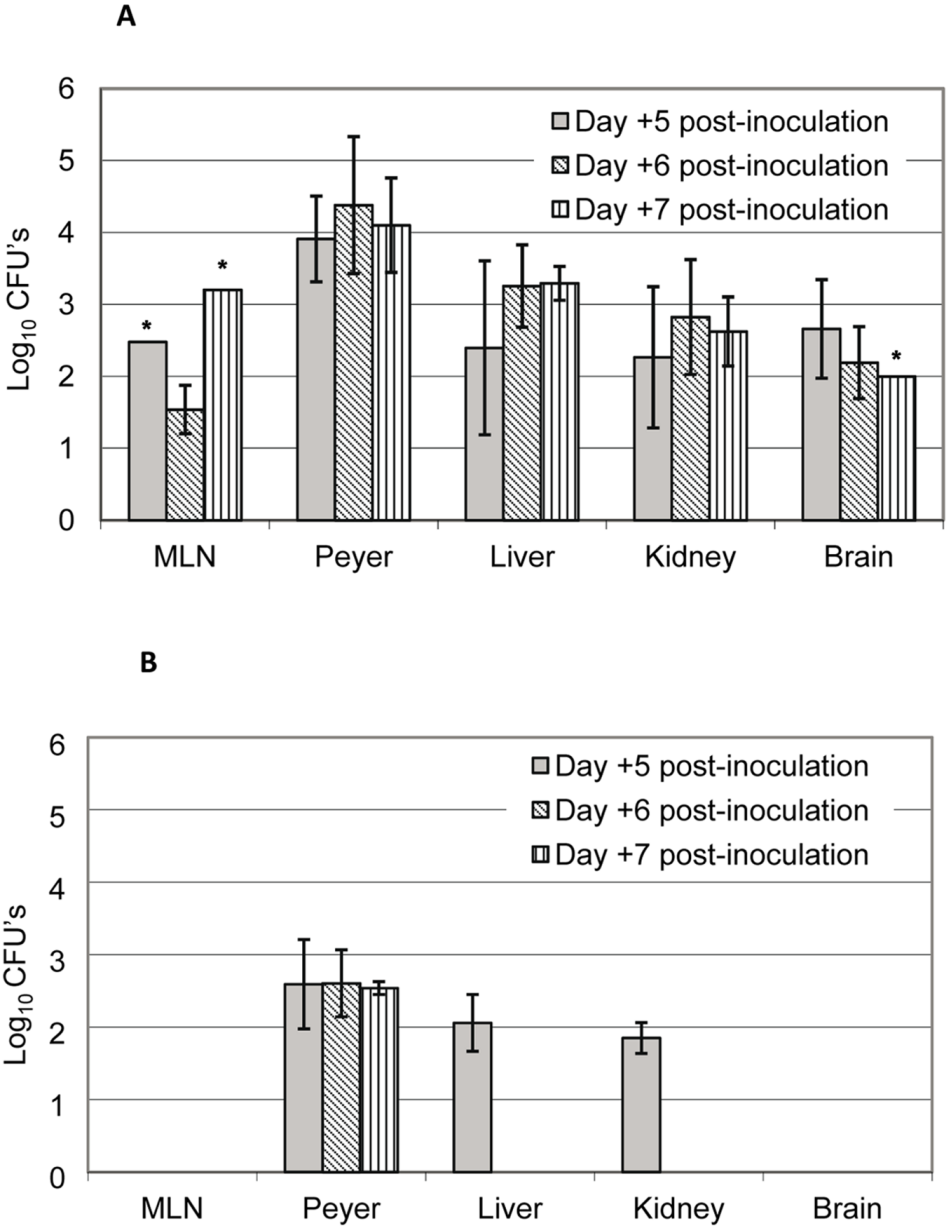
Translocation and dissemination from the gut of strains D14 and D23 after oral inoculation. Burdens of dietetic strains D14 (A) and D23 (B) are shown after oral administration of ICR/Swiss mice receiving antibiotic supplementation in the drinking water and immunosuppressant intraperitoneally. Each bar represents polled data from 2 to 4 mice and exceptionally from 1 mouse (asterisk). Results are expressed as the mean ± SD.

One day after gastric inoculation (day 1), only mice inoculated with the D14 strain presented ruffled hair, symptom of an infection process, but retained good mobility and experienced no weight loss. These characteristics were maintained for the remainder of the experiment, being more pronounced in the last days. First, we evaluated the translocation of D14 and D23 from the intestinal tract by assessing burden levels in Peyer’s patches; both isolates crossed the intestinal barrier in the experimental conditions although translocation was more consistent in the case of the virulent isolate (D14) as it was detected in Peyer's patches from 100% of mice tested, while the avirulent isolate (D23) was detected in 75% of them (Table 6). Remarkably, burden levels of isolate D14 in this organ were higher than those of isolate D23 and these differences were statistically significant (*p*<0.05, one-way ANOVA analysis) (Figure 12). In regard to dissemination, differences between both isolates were evident. D14 spread to MLNs in 33% of mice and to distant organs (livers, kidneys and brains) in 75%. Conversely, D23 was not found in MLNs or brains and was occasionally found in livers and kidneys (in 25% of total mice and only at day 5 post-inoculation).

**Table 6:** Intestinal translocation and dissemination of isolates D14 and D23 based on the number of mice that showed yeast burdens in target organs.

	No. of mice with D14/total sacrificed mice (%)			No. of mice with D23/total sacrificed mice (%)		
Day ofsacrifice(a)	Peyer’spatches	MLNs(b)	Otherorgans(c)	Peyer’spatches	MLNs(b)	Otherorgans(c)
Day 5	4/4 (100)	1/4 (25)	3/4 (75)	3/4 (75)	0/4 (0)	3/4 (75)
Day 6	4/4 (100)	2/4 (50)	3/4 (75)	4/4 (100)	0/4 (0)	0/4 (0)
Day 7	4/4 (100)	1/4 (25)	3/4 (75)	2/4 (50)	0/4 (0)	0/4 (0)
**Total**	**12/12 (100)**	**4/12 (33)**	**9/12 (75)**	**9/12 (75)**	**0/12 (0)**	**3/12 (25)**

(a) Day of sacrifice after initiation of oral administration of the yeast.

(b) MLNs: mesenteric lymph nodes.

(c) Other organs: brain, kidneys, liver.

### 6. Identity of the Yeast Cells Recovered from Infected Mice

In order to confirm that the colonies recovered from the infected mice really belonged to the yeast strain inoculated, mtDNA restriction analysis was performed both in the intravenous and oral models of infection.

The mtDNA pattern of each recovered colony form each organ was compared with that displayed by the strain inoculated in each mouse, and 100% of identity was found in all cases (data not shown).

## Discussion

In recent years, the perception that *S. cerevisiae* as harmless has changed due to an increase in the number of infections caused by this yeast [6], [59], [60]. There is no doubt about the opportunistic nature of these infections; however, there are studies indicating that not all strains are capable of developing infection, even though favorable conditions are present [15], [16], [31]. Regarding *S. cerevisiae* strains contained in dietary supplements, it is unknown whether they have intrinsic properties that could contribute to virulence in humans. Given that these commercial preparations constitute the main route by which living *S. cerevisiae* cells gain entry into the human body, the study of their *in vivo* virulence is of great importance.

When the systemic virulence of dietetic *Saccharomyces* isolates was assessed by intravenous inoculation, we found that among the isolates tested D2, D4 and D14 were able to lodge and persist in the analyzed organs (brains and kidneys) while isolate D23 was undetectable. The ability to lodge has previously been described for a large majority of *S. cerevisiae* clinical isolates [15], [16], [31], [37] and for some non-clinical isolates [16], [17], [31], [37]. Burden values obtained for our isolates were higher or similar to those observed for the positive control (YJM128) although this strain was previously described as highly virulent in an immunocompetent murine system (CD-1) [16]. This is probably due to the fact that a yeast strain can exhibit different degrees of virulence depending on the kind of mouse used to mimic the systemic infection [15]. Indeed, YJM128 was more virulent in DBA/2N mice than in CD-1 mice [15], [16] and, in the light of our results, it appears to be less virulent in BALB/c mice. Moreover, a loss of virulence with the passage of time cannot be ruled out, as described by Clemons *et al.* [16] for another isolate, and it must be taken into account that YJM128 was first described in 1994. Despite this observation, we have referred to YJM128 as a positive control of virulence when intravenous inoculation was used as the infection route.

One of the most remarkable results of this work was the ability of a strain isolated from a commercial dietary supplement (the D14 strain) to cause mortality in BALB/c mice. This result confirms previous data reported by our group indicating a remarkable virulence of this strain using other mouse strains (DBA/2 and C3H/HeN) [37]. The null mortality observed in one of the infection assays with D14 could be a result of increased resistance to infection associated with a mature immune system in this particular batch of mice. In a previous study, we have shown the influence of immune system maturity on the virulence of *S. cerevisiae* strains when BALB/c mice were used [31].

By contrast, isolates D2, D4 and D14 produced clear signs of disease in mice, like the YJM128 strain. The same signs of disease were described in the course of *C. albicans-*related infections, a typically pathogenic yeast strain [61]. Therefore, this information would indicate that these *S. cerevisiae* isolates not only can survive in mouse blood and then spread to different organs, but they are also able to trigger a systemic infection. This finding is important because colonization is not always connected with symptoms [31] and highlights the particularity of these isolates.

Once host-yeast interaction takes place, the expression of virulence factors and the ability to proliferate and survive in the host or to cause damage could differ from *in vitro* conditions. That is why it is questionable whether there is an association between the phenotypic traits expressed by the isolates and their virulence *in vivo*. In our study, we have found data suggestive of a correlation between some of the virulence-related traits *in vitro* and the results obtained *in vivo* in a murine model of i.v. inoculation, since the strains thought potentially pathogenic based on phenotypic traits (D2, D4 and D14) were able to lodge and persist for some time in the analyzed organs and developed symptoms of infection. Moreover, the isolate D23, which only showed a low phospholipase activity among the virulence-related traits assayed, was found to be completely avirulent *in vivo*.

We conducted a statistical analysis including all the strains whose *in vivo* virulence was tested (dietetic strains and control strains) to evaluate this hypothesis (Table 7) and we found that the ability to grow at 39°C and pseudohyphal growth are positively and very strongly associated with *in vivo* virulence in BALB/c mice (Spearman’s correlation coefficient = 1, *p*<0.01; similarity values = 1, Jaccard Proximities test). These traits have previously been studied in clinical and non-clinical *S. cerevisiae* strains [28] and have been associated with the *in vivo* virulence of *S. cerevisiae* [11], [31]. Some considerations can be made in relation to growth at >37°C. We noticed that the association between the ability to grow at 42°C and the *in vivo* virulence was in the boundaries of significance (*p* = 0.052, Spearman’s correlation coefficient, Table 7) suggesting that both traits are close. This result resembles that shown by McCusker *et al.* [11] although the latter author reported a strong association between the ability to grow at 42°C and the ability to colonize immunocompetent CD-1 mice. This discrepancy is probably due to the type of strains they tested. They analyzed both clinical and non-clinical strains and show that the ability to grow at 42°C was mainly related to the clinical origin. Indeed, it seems logical that microorganisms with difficulties to grow at human body temperature or those reached during febrile states, are not able to develop systemic infection, as observed for strains D6 and D23. The inability of these strains to grow at temperatures ≥37°C is probably related to their hybrid nature and habitat since *S. cerevisiae-S. bayanus* and *S. cerevisiae-S. kudriavzevii* hybrids are primarily associated with alcoholic fermentations carried out at low temperatures [62]. Therefore, it seems unlikely that the hybrid nature facilitates colonization, invasion and dissemination during a systemic infection. The opposite occurs at the industrial level, since hybrids are better adapted than their parental strains to resisting stress during fermentation processes [41]. According to these comments hybrids could be safer than *S. cerevisiae* strains for the preparation of dietary supplements. However, it would be necessary to extend this study to a larger number of hybrid isolates to confirm this hypothesis.

**Table 7:** Statistical analysis of association between virulence-associated phenotypic traits and *in vivo* virulence.

	*In vivo* virulence^b^	
Traits tested^a^	Jaccardproximities^c^	Spearman’s Rho(significance)^d^
Growth at 39°C	1.000	1.000 (*p*<0.01)
Pseudohyphal growth	1.000	1.000 (*p*<0.01)
Protease activity	0.800	0.730 (*p* = 0.062)
Growth at:		
42°C	0.750	0.750 (*p* = 0.052)
37°C	0.667	0.471 (*p* = 0.286)
Phospholipase activity	0.600	0.417 (*p* = 0.352)
Switching	0.600	0.417 (*p* = 0.352)
Plastic adherence	0.500	0.548 (*p* = 0.203)
Invasive growth	0.250	0.354 (p = 0.437)
MAPK activation:		
Slt2	0.250	0.354 (*p* = 0.437)
Kss1	0.000	n.c.

a Traits ordered by proximity for better comprehension. Data used in the case of strains isolated from dietary supplements are shown in Table 5. Phenotypic traits of control strains, simplified following the codes in Table 5, are: YJM128 (+, +, +, +, –, +, +, n.d., n.d., n.d., n.d.), W303 (+, –, –, +, –, –, –, n.d., n.d., n.d., –), CECT 10.431 (+, –, –, –, –, –, –, n.d., n.d., n.d., n.d.). Data of YJM128 have been obtained previously [28]. “+” and “–” were coded numerically as “1” and “0” respectively for statistical analysis.

b *In vivo* virulence determined by intravenous inoculation of strains D2, D4, D14, D23, YJM128, W303 and CECT 10.431 in BALB/c mice. Virulence was simplified as follows: high burdens levels in brain (3.18 to 5.31 mean log_10_ CFU) at 7 and 15 days post-infection were considered + and lower burdens levels and the inability to colonize was considered –.

c A proximity value of “1” indicates the maximum association and value “0” indicates the opposite. Jaccard proximities were calculated excluding joint absences from consideration.

d Values of “1” indicates perfect correlation and “0″ indicates no relationship.

n.c. Spearman’s correlation cannot be calculate because this trait shows constant values (see Table 5).

Protease and phospholipase production, invasive growth and colony switching, have been associated with clinical origin but they are less obvious virulence-related attributes [12], [16], [28], [63]. This last statement is consistent with our statistical analysis of association (Table 7). We did not observe any significant association in the case of MAPK activation and plastic adherence either, even though both traits are important pathogenic factors in *C. albicans* [25], [26], [27]. It is worth commenting that the increased phosphorylation of the MAPK Slt2 would reflect an activation of the CWI pathway in the virulent D14 strain that could be due to an altered cell wall. The alteration of this external structure in yeast cells might affect immune system recognition, as proposed for a *S. cerevisiae* hypervirulent mutant defective in the *SSD1* gene [64]. These authors suggest that the altered cell surface of this mutant might lead both to misrecognition by the innate immune system and induce shock in the mice by overstimulation of the proinflammatory response. Furthermore, the surface-adhering ability observed in D2 and D4, with intermediate virulence level after intravenous inoculation, could facilitate their entry to patients’ bloodstream and internal organs and would, therefore, make them more infectious in theory than other strains lacking this ability. Strain D5 (Ultra-Levura) showed the lowest adherence to any of the plastics tested among the analyzed strains This was unexpected given given its virulence in experimental models [17], [31] and its association with human infection via contamination of central venous catheter by the colonized hands of health-care workers [6], [59]. In constrast, the *S. cerevisiae* clinical isolate 102 showed significant plastic adhesion ability, comparable to *C.albicans* for polyurethane catheters and ever higher to that displayed by this pathogenic species to polystyrene.

As we have stressed, there are *Saccharomyces* isolates from dietary products able to colonize and disseminate and even cause death in animal models when inoculated directly into the blood. However, since these yeast-cell-containing products are ingested orally, infection models derived from translocation across the gastrointestinal mucosa are required to determine which strains may pose a real potential safety risk to humans. To date, the few studies into the occurrence of *S. cerevisiae* intestinal translocation are limited to investigating its probiotic properties; in particular the protective capacity against infection by pathogenic bacteria and rotavirus [65]–[70]. There is only one study investigating the virulence of *S. cerevisiae* when administered as a probiotic [71]. Given that isolate D14 was highlighted for its virulent potential in an i.v. inoculation model, we considered it important to assess the negative impact of its consumption. In clinical cases of fungemia derived from the intake of live *S. cerevisiae* cells, in particular *S. boulardii*, it has been postulated that translocation from the gut to the bloodstream occurs in debilitated patients with impaired gastrointestinal mucosa, treatment with broad-spectrum antibiotics and/or severe immunosuppression [34], [35], [72], [73]. The same behavior can be expected in the case of *S. cerevisiae* isolates from dietary supplements, justifying the use of animal systems that mimic the abovementioned conditions. Moreover by using an *in vitro* intestinal epithelial barrier model, we have previously shown that, in contrast to *C. glabrata* and *C. albicans*, *S. cerevisiae* was not able to cross the intestinal barrier unless its integrity was compromised [74]. Thus, a murine model has been established using mice pretreated with a combination of three antibiotics (streptomycin, gentamicin and bacitracin) and the administration of high-doses of dexamethasone, which has enabled intestinal infectivity of isolate D14 to be studied and compared with the isolate considered avirulent (D23).

The burden levels detected in Peyer's patches, the first organ where the pathogenic agents crossing the gut wall can be phagocyted, show the occurrence of intestinal translocation in sub-mucosa in both isolates, although it was more consistent for D14. Translocation has already been described to occur in probiotic strains of *S. cerevisiae* during studies of its immunomodulating action or competition with harmful bacteria and rotavirus [65]–[70]. In some of these studies, the presence of yeast was detected in mesenteric nodes of immunocompromised mice [68], young mice (21 days) [69], [70] or infant mice [65], [66]. However, studies investigating the spread of yeast cells to distant organs did not report the presence of yeast cells in such organs [70], [71] or their presence was inconsistent and occasional [65], [68]. These negative results were probably due to the use of inappropriate murine model that did not combine immunosuppression and intestinal trauma. In our study, a four-day application of dexamethasone, a drug that leads to immunosuppression, besides damaging the gastrointestinal mucosa, allowed D14 to spread to mesenteric nodes and distant organs. The fact that some mice showed no burdens in mesenteric nodes but in distant organs could indicate the occurrence of direct vascular invasion with subsequent yeast transport through the portal system to the liver and, hence, to other distant organs besides spreading via the lymphatic system. The results obtained for isolate D14 contrast with the behavior of D23 and agree with the virulence degree assigned to both isolates after the i. v. infection study. Therefore, in the presence of conditions promoting intestinal translocation, the spread to internal organs appears to be highly strain-dependent and only seems to occur when it possesses innate virulence attributes; however, we cannot generalize since only two strains have been tested. This is in agreement with a study using piglets orally inoculated with *C. albicans,* in which a non-virulent strain showed poorer dissemination than a virulent one [75].

To our knowledge, this study is the first to demonstrate the occurrence of virulence-related traits in *Saccharomyces* strains ingested as living cells in the form of commercial dietary products. Moreover, we show that some of these isolates can be pathogenic to animal models when inoculated intravenously and at least one of them, when the inoculation route is oral and the mice have an impaired gastrointestinal mucosa. Taking into account that dietary products could be a natural way for live yeast cells to gain entry into the human body, the strains used in their production should be avirulent. Therefore, some recommendations should be made to yeast production and distribution companies and dietary supplement manufacturers. On the one hand, prior screening of virulence-related attributes, such as those tested here (particularly growth at 39°C and pseudopyphal growth), appears to be necessary when selecting new strains. On the other hand, the health foods currently available on the market should also be checked to ensure their safety. All these precautions are especially important for immunocompromised individuals. Furthermore, the availability of virulent and avirulent isolates is very useful for future works aimed to explore the mechanisms involved in *S. cerevisiae* virulence as well as in other emerging food-borne yeast pathogens.

## Supporting Information

Figure S1 **Example of colony phenotype switching**. Representative colonies of D2 (A) and D14 strain (B) after five days at 30°C on YPD with phloxine B.
(TIF)
