# Supplementary figures and images for: Pathogenic Potential of Saccharomyces Strains Isolated from Dietary Supplements
Source: PLoS One. 2014 May 30;9(5):e98094. doi: 10.1371/journal.pone.0098094 (PMC4039445; doi:10.1371/journal.pone.0098094)

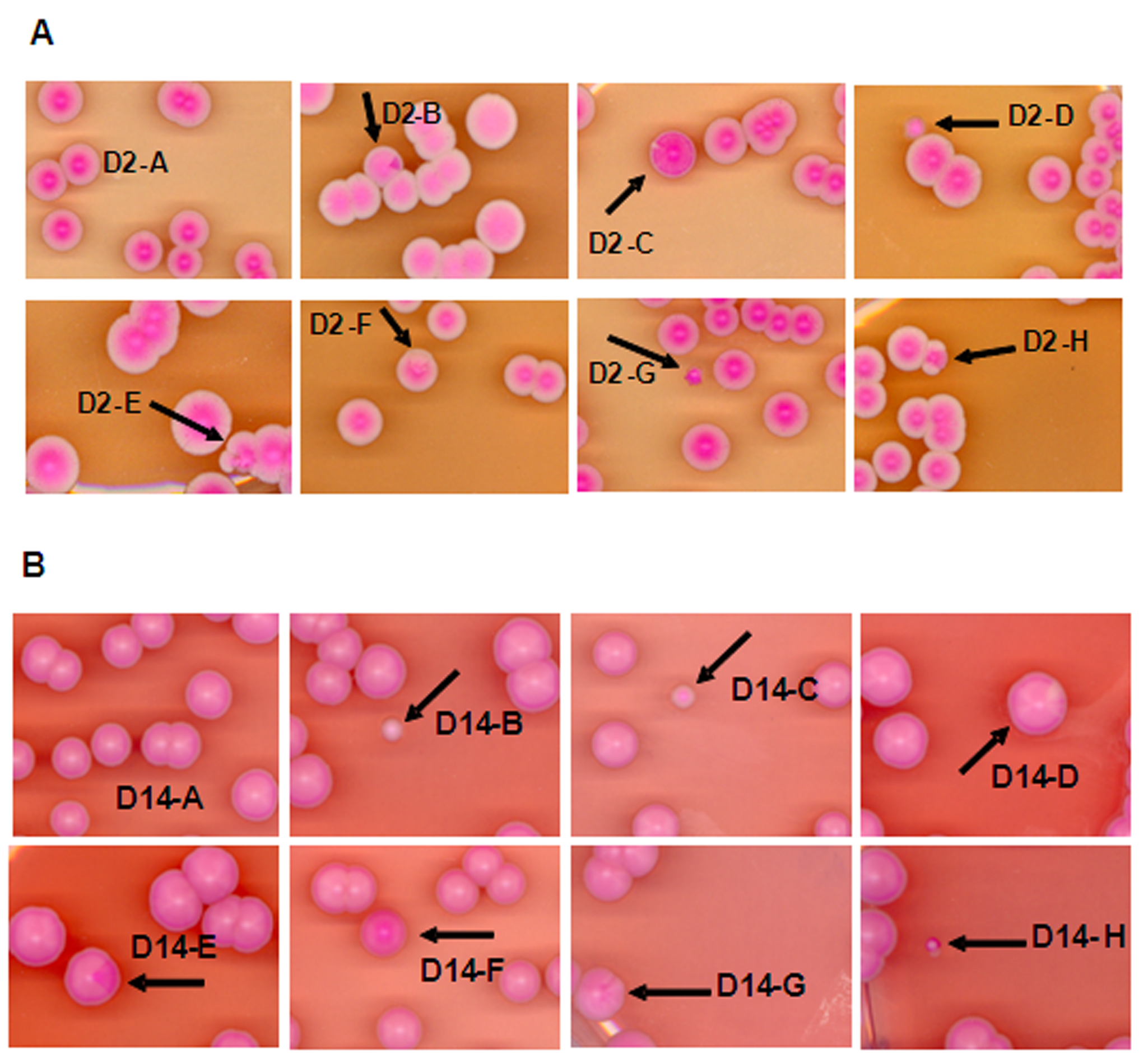

Supplement: Figure S1 — Example of colony phenotype switching. Representative colonies of D2 (A) and D14 strain (B) after five days at 30°C on YPD with phloxine B. (TIF) [file pone.0098094.s001.tif]
